# The within-host population dynamics of *Mycobacterium tuberculosis* vary with treatment efficacy

**DOI:** 10.1186/s13059-017-1196-0

**Published:** 2017-04-19

**Authors:** Andrej Trauner, Qingyun Liu, Laura E. Via, Xin Liu, Xianglin Ruan, Lili Liang, Huimin Shi, Ying Chen, Ziling Wang, Ruixia Liang, Wei Zhang, Wang Wei, Jingcai Gao, Gang Sun, Daniela Brites, Kathleen England, Guolong Zhang, Sebastien Gagneux, Clifton E. Barry, Qian Gao

**Affiliations:** 1 Department of Medical Parasitology and Infection Biology, Swiss Tropical and Public Health Institute, 4002 Basel, Switzerland; 2 University of Basel, 4001 Basel, Switzerland; 3 Key Laboratory of Medical Molecular Virology of Ministries of Education and Health, Institutes of Biomedical Sciences and Institute of Medical Microbiology, School of Basic Medical Sciences, Fudan University, Shanghai, 200032 China; 4 Tuberculosis Research Section, Laboratory of Clinical Infectious Diseases, NIAID, NIH, Bethesda, MD 20892 USA; 5 Institute of Infectious Disease and Molecular Medicine, and the Department of Clinical Laboratory Sciences, Faculty of Health Sciences, University of Cape Town, Rondebosch, 7701 South Africa; 6 Henan Provincial Chest Hospital, Zhengzhou, 450003 Henan China; 7 Sino-US International Research Centers of Tuberculosis, Zhengzhou, 450003 Henan China; 8 Henan Public Health Clinical Center, Zhengzhou, 450003 Henan China

**Keywords:** Tuberculosis, Within-host evolution, Combination therapy, Drug resistance, Whole genome sequencing

## Abstract

**Background:**

Combination therapy is one of the most effective tools for limiting the emergence of drug resistance in pathogens. Despite the widespread adoption of combination therapy across diseases, drug resistance rates continue to rise, leading to failing treatment regimens. The mechanisms underlying treatment failure are well studied, but the processes governing successful combination therapy are poorly understood. We address this question by studying the population dynamics of *Mycobacterium tuberculosis* within tuberculosis patients undergoing treatment with different combinations of antibiotics.

**Results:**

By combining very deep whole genome sequencing (~1000-fold genome-wide coverage) with sequential sputum sampling, we were able to detect transient genetic diversity driven by the apparently continuous turnover of minor alleles, which could serve as the source of drug-resistant bacteria. However, we report that treatment efficacy has a clear impact on the population dynamics: sufficient drug pressure bears a clear signature of purifying selection leading to apparent genetic stability. In contrast, *M. tuberculosis* populations subject to less drug pressure show markedly different dynamics, including cases of acquisition of additional drug resistance.

**Conclusions:**

Our findings show that for a pathogen like *M. tuberculosis*, which is well adapted to the human host, purifying selection constrains the evolutionary trajectory to resistance in effectively treated individuals. Nonetheless, we also report a continuous turnover of minor variants, which could give rise to the emergence of drug resistance in cases of drug pressure weakening. Monitoring bacterial population dynamics could therefore provide an informative metric for assessing the efficacy of novel drug combinations.

**Electronic supplementary material:**

The online version of this article (doi:10.1186/s13059-017-1196-0) contains supplementary material, which is available to authorized users.

## Background

The public health and economic impact of drug resistance is steadily increasing [1, 2]. Empirical studies and mathematical modeling strongly support the simultaneous use of multiple drugs with disparate mechanisms of action—combination therapy—as a powerful approach to enhance treatment efficiency and reduce the likelihood of resistance [3–5]. The reason why combination therapy is so effective partially stems from the constraint placed on cells to acquire sufficient mutations to overcome the pressure from multiple drugs [6, 7]. Combination therapy has therefore become the cornerstone of treatments for some of the major causes of human mortality and morbidity: human immunodeficiency virus (HIV) infection, malaria, tuberculosis (TB), and cancer. Nonetheless, drug resistance remains a serious threat to the success of treatment despite the broad implementation of combination therapy [8–10]. Less effective drug combinations [11], de facto monotherapy due to differences in drug penetration and stability [12–14], poor drug quality [15], prior existence of resistance [16, 17], and poor patient compliance [18] can all result in the alleviation of drug pressure. In cases where resistance is mediated by chromosomal mutations, which includes HIV, TB, malaria, and cancer, decreased drug pressure can facilitate emergence of resistance mutations leading to multidrug resistance [19–23]. The evolutionary processes driving drug resistance occur across several scales, spanning from the de novo emergence of resistance mutations to their transmission and ultimate fixation within a population [24]. The ability to integrate information across scales would improve our understanding of the adaptive processes involved [25] and result in enhanced approaches to combination therapy.

Directly observed treatment, short course (DOTS) is the current standard treatment for drug-susceptible TB. DOTS relies on a combination of four antibiotics—isoniazid, rifampicin, ethambutol, and pyrazinamide—administered with supervision for a total of 6 months. When implemented correctly, DOTS is very effective; it reduces the rate of disease relapse to less than 5% [26] and the rate of acquired resistance to similarly low levels [27, 28]. The two most effective drugs in DOTS are isoniazid and rifampicin. Resistance to both is defined as multidrug-resistant TB (MDR-TB) and is associated with a poorer prognosis and longer treatment times [29]. Despite the efficacy of DOTS, MDR-TB has emerged independently on multiple occasions across the globe [30–32]. Repeated failure to optimally administer the combination treatment culminates in the emergence of strains resistant to all constituents of the regimen [23, 33, 34]. Whole genome sequencing (WGS) has been indispensable for identifying the mutations that underpin drug resistance [35–37]. Placing WGS data in the context of evolutionary theory allows the detection of treatment-driven positive selection of resistance determinants both as convergent evolution in epidemiologically unrelated populations [35, 38–40] as well as enrichment and ultimate fixation of resistance-conferring alleles within single patients during the course of treatment [41–45].

However, the focus on treatment failure presents a biased view of the evolutionary processes involved: 95% of all TB patients infected with fully drug-susceptible strains are successfully treated [26]. As a result, we do not, at present, fully understand what selective forces shape populations under successful treatment. It is unclear how treatment efficacy impacts the dynamics of bacterial populations within the host and ultimately how it affects clinical outcomes. Moreover, we do not know whether successful drug treatment leads to the enrichment of mutations that may be beneficial to the bacterium during future treatments. The significance of this possibility could explain the fact that resistance rates are highest among TB patients that have previously been treated [26]. We therefore sought to understand the impact of treatment efficacy on TB from the perspective of population genetics, to try to understand how close we come to resistance every time we treat.

We explored the nature of selective forces that shape *Mycobacterium tuberculosis* complex (MTBC) populations by investigating their dynamics within the human host. We used an approach based on very deep population WGS (approximately 1000-fold read coverage per site) of serial sputum isolates from TB patients with high bacillary loads undergoing treatment. We report that MTBC populations in the human host are genetically more dynamic than previously thought. Furthermore, the presence and extent of drug pressure influences the observed changes. Our findings shed light on the genetic principles that underpin well-established clinical practices: combination therapy based on at least four effective drugs constrains the adaptive landscape of MTBC through purifying selection. Conversely, treatment with fewer than four effective drugs alleviates this constraint, allowing positive selection of resistance determinants.

## Results

### Sampling of bacterial populations in the host

**Fig. 1:**
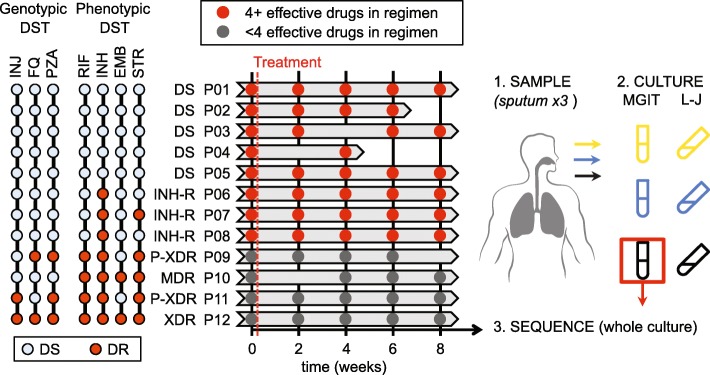
Characteristics of the study population. Our study was based on serial sputum isolates obtained from 12 TB patients at 2-week intervals. We obtained three sputum samples at each time point and cultured each on Löwenstein–Jenssen solid medium (*L-J*) or in a mycobacterial growth indicator tube (*MGIT*); we chose one culture per patient per time point for deep sequencing. Eight patients (P01–P08) were treated with a combination composed of at least four effective antibiotics (sampling indicated by *red circles*). While four patients (P09–P12) were treated with fewer than four effective antibiotics (*grey circles*). Phenotypic drug susceptibility testing (*Phenotypic DST*) and genotypic drug susceptibility testing (*Genotypic DST*) results are shown for each patient with *light blue dots* indicating drug susceptibility (*DS*) and *red dots* reflecting drug resistance (*DR*). The antibiotics are abbreviated as: *RIF* rifampicin, *INH* isoniazid, *EMB* ethambutol, *STR* streptomycin, *INJ* injectable aminoglycosides, *FQ* fluoroquinolones, *PZA* pyrazinamide. Resistance profiles of strains are given as: *DS* drug susceptible, *INH-R* isoniazid monoresistant, *MDR* multidrug resistant, *P-XDR* pre-extensively drug resistant, *XDR* extensively drug resistant. MDR is defined as RIF and INH resistant, XDR is MDR with additional resistance to FQ and INJ, and P-XDR is MDR with either FQ or INJ resistance

We collected sputum samples from 12 TB patients at entry, 2, 4, 6, and 8 weeks after commencement of treatment. Three sputum samples were obtained at each time point for each patient. The resistance profile of the initial MTBC isolates was determined with standard phenotypic drug susceptibility testing (Additional file 1: Table S1) and is summarized together with the frequency of sampling in Fig. 1. As treatment progressed, bacterial loads in sputum decreased at varying rates, leading to variation in the number of culture-positive samples we obtained from each patient. The composition of the drug combination given to each patient differed based on the available information on the resistance profile of the infecting bacteria and the judgment of the treating physician (Additional file 1: Table S2).

Treatment guidelines provided by the World Health Organization [46, 47] state that patients should receive a combination of at least four effective antibiotics. Based on these recommendations, we could assign patients to one of two groups: patients 1–8 received four or more (4+) effective drugs, while patients 9–12 received fewer than four effective drugs. This grouping reflected the resistance profiles of infecting strains as well, since all patients receiving fewer than four drugs were also infected with highly resistant strains. The efficacy of treatment was reflected in the rate of bacterial clearance. We used time to culture positivity as a proxy for intra-patient bacterial burden in a regression analysis. As expected, we observed a significant reduction in bacterial burden over time in patients who received at least four effective drugs (time to positivity increased by 1.15 days per week of treatment, *p* < 0.001). The rate of load reduction was significantly lower in patients receiving fewer than four effective drugs (time to positivity slowed to increase by 0.32 days per week of treatment, *p* < 0.05; Additional file 1: Figure S1; see Additional file 2: Table S6 for raw data).

Our goal was to gain a comprehensive view of the overall heterogeneity of MTBC populations within the host lung and follow their dynamics over time. To achieve this, we performed deep sequencing of the entire bacterial population recovered from sputum isolates (see Additional file 1: Table S3 for sequencing depth estimates). We focused our analysis on single nucleotide polymorphisms (SNPs). For technical reasons we did not explore the impact of small insertions and deletions or gross chromosomal alterations such as duplications of genomic regions. Structural discrepancies between the strains included in our study and the reference genome could give rise to some, albeit small, proportion of the variation we detect. While duplications of genomic regions have been shown to occur in MTBC [48, 49], they are relatively rare; we therefore expect the impact of such variation to be relatively small. We then devised a noise-filtering algorithm to minimize the impact of sequence heterogeneity introduced by culture, PCR, sequencing, and mapping errors. We calibrated the filtering parameters by incorporating information about the error profiles from simulated sequencing reads and sequencing data from individual MTBC colonies expanded in growth medium (Additional file 1: Figures S2–S4; and Additional file 1: Section 2). We defined two types of SNPs in our samples: intra-host fixed SNPs (f-SNP) where all of the sequencing reads from a population supported a base that is different from the reference; and intra-host variable SNPs (v-SNP) where only a fraction of the reads supported a base that was different from the reference, while the remaining reads were consistent with the reference. All variable positions had only two alleles—wild type and mutant.

The number of f-SNPs was constant over time in most patients (Additional file 1: Table S4). The exception was patient 11, where there was an apparent decrease in f-SNPs at week 6. This patient experienced a cavitation of a large granuloma during the course of treatment, which may have led to a transient change in the major clone found in sputum resulting in what were f-SNPs from the dominant clone to decrease in frequency and become v-SNPs [50]. Overall, we detected 492 v-SNPs that fulfilled our criteria across all the patients and time points. The number of detected v-SNPs decreased slightly with decreasing bacterial loads within patients, but was not biased by sequencing depth (Additional file 1: Figure S6). The observation that treatment decreased the overall heterogeneity of the population is in line with the expectation for a dying population (Additional file 1: Section 3; Additional file 1: Figure S5). Importantly, the number of v-SNPs varied between serial samples from each patient, reflecting bacterial population dynamics in the lung (Additional file 1: Figure S7). Taken together, these findings suggest that sequencing errors were unlikely to contribute to v-SNP heterogeneity, allowing for the biological interpretation of our data. Complete lists of v-SNPs and f-SNPs are available in Additional file 3: Table S7 and Additional file 4: Table S8, respectively.

### Sputum samples are heterogeneous

MTBC is normally confined within multiple spatially segregated anatomical structures called granulomas. Granulomas are dynamic structures resulting from an orchestrated immune response to MTBC infection and are located in the lung or adjacent lymph nodes. The relationship between bacterial populations in granulomas and those in the sputum is not well understood; however, it is widely accepted that granulomas with access to airways serve as the source of bacteria in the sputum [51]. As a result, the transition of MTBC from granuloma to sputum is likely to be a stochastic process. We therefore reasoned that analysis of several sputa obtained from the same patient within a short time span would give us an indication of sampling consistency.

**Fig. 2:**
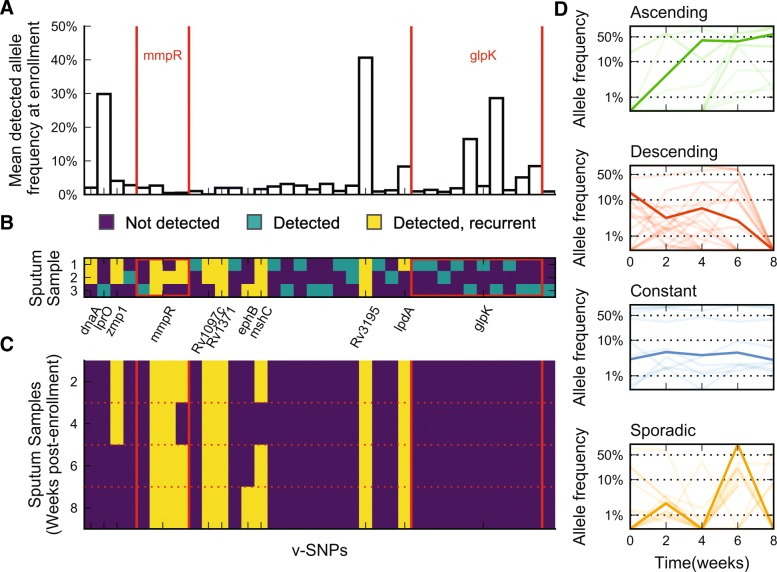
Sputum samples under-represent the true genetic diversity of MTBC populations in the lung. We sequenced three samples from the enrollment time point of patient 12 and compared the detected population heterogeneity. **a** Mean frequency of detected v-SNPs across samples. Four v-SNPs affecting Rv0678 (*mmpR*) and ten v-SNPs affecting Rv3696c (*glpK*) are marked with *red lines*. **b** Detection pattern of v-SNPs across the three sputum samples. v-SNPs were classified as recurrent if they were detected in at least one sputum sample from a later time point. **c** Temporal detection pattern for listed v-SNPs across sputum samples isolated from patient 12 2, 4, 6, and 8 weeks post-enrollment. **d** Patterns of v-SNP temporal dynamics detected across all patients. One trajectory per type is highlighted for illustration purposes

We sequenced three sputum samples obtained from patient 12 on the day of enrollment to address sampling consistency (Fig. 2a–c). We detected 36 v-SNPs in total across the three parallel sputum samples; only four of these were detected in all samples, and five were shared by two separate samples. The remaining 27 alleles (75%) were unique to each sample, indicating that we are likely to routinely underestimate the true heterogeneity of the MTBC population in a patient’s lung. Interestingly, the distributions of allele frequencies of shared and unique v-SNPs were not significantly different (Mann–Whitney U-test, *p* = 0.86), suggesting that observing the same v-SNP across multiple samples was not simply a function of the estimated v-SNP abundance. We then used data from later time points to determine what proportion of the v-SNPs identified in enrolment samples were repeatedly detected and classify them as recurrent. The majority of variants that were detected in more than one parallel sample were also recurrent (highlighted in yellow in Fig. 2b), showing that we are re-sampling part of the heterogeneity, allowing for biological inferences.

Looking at the identity of the affected genes, we found that two contained multiple v-SNPs: *mmpR* (Rv0678) and *glpK* (Rv3696c) contained four and ten v-SNPs, respectively. The former is a known mediator of clofazimine and bedaquiline cross-resistance [52], while the later was shown to be essential for growth on glycerol, but dispensable in the mouse model of infection [53]. Most of the *mmpR* v-SNPs accounted for a very small proportion of the overall population (1–5% of the population) but were nonetheless mostly stable over time—recurrent. *glpK* variants on the other hand were all unstable despite some of them being relatively abundant in some samples, accounting for 20–30% of the population. In fact, we did not observe any difference in variant frequency between recurrent and unstable v-SNPs in the parallel samples from patient 12 (Mann–Whitney U-test, *p* = 0.24), again suggesting that the temporal stability of variants was not simply a function of abundance, but rather a reflection of an ongoing biological process. Furthermore, this also provides evidence of the co-existence of separate populations of MTBC within a host.

Quantifying the relative abundance of individual alleles in sputa at different times during treatment therefore allows us to approximate the changes in the composition of bacterial populations within each patient. A naïve overview of temporal dynamics of recurrent v-SNPs revealed four types of allele trajectories: ascending, descending, constant, and sporadic (Fig. 2d), showing that MTBC populations within the host are both dynamic and heterogeneous.

### Intra-host heterogeneity is driven by very rare variants

**Fig. 3:**
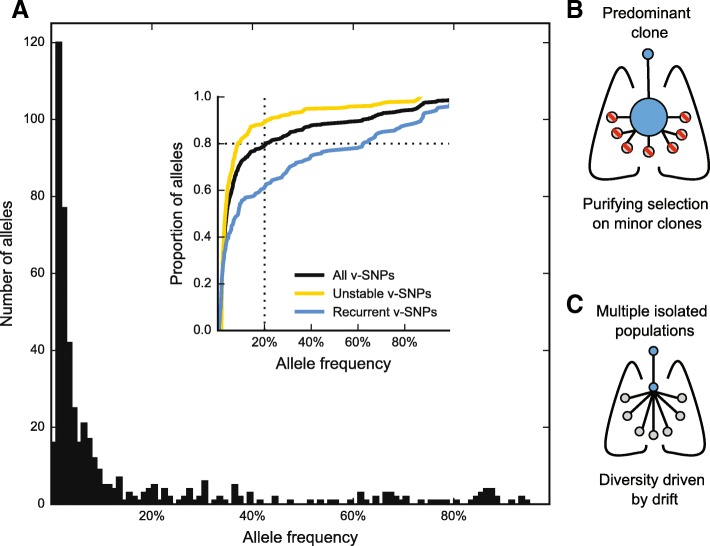
Structure of MTBC populations in TB patients. **a** Folded site frequency spectrum: a histogram of estimated variable allele frequencies within MTBC populations in TB patients. Cumulative distributions of allele frequencies for all variable SNPs (v-SNPs) are shown in *black*—80% of all the v-SNPs are present at an estimated frequency of less than 20% (*dotted line*). The corresponding distributions for v-SNPs that were detected in sputa from a single time point (unstable, *yellow*) or from multiple time points (recurrent, *blue*) are also shown. The observed distribution of alleles could arise from **b** a dominant clone of MTBC colonizing the lung and minor genetic variants continuously emerging from it which are selected against by purifying selection. Alternatively, **c** a large number of physically separated populations each produce minor variants. In this setting selection would be less efficient and population dynamics would be driven by genetic drift

We started with a general analysis of population heterogeneity within our isolates by combining all the detected v-SNPs into a folded site frequency spectrum (SFS; Fig. 3a). The intra-host SFS had a leptokurtic distribution with a strong positive skew. The majority of the detected sequence heterogeneity therefore resulted from an abundance of rare alleles in the population—approximately 80% of the total heterogeneity was accounted for by v-SNPs with a frequency of less than 20%. This degree of variation is higher than reported previously [54–56] and shows that MTBC might explore its mutational space to a greater extent than previously thought. Importantly, the intra-patient SFS was very similar to the inter-host SFS reported by Pepperell and colleagues [57], suggesting that forces shaping the diversity at the host population level are already prominent within patients, before the bottleneck of transmission.

Unlike the comparison of frequency distributions at the initial time point in patient 12, which showed no difference between frequencies of recurrent and unstable v-SNPs, expanding the analysis to the whole sample set showed that, overall, recurrent v-SNPs occurred at higher frequencies in the sampled populations. This is illustrated by the cumulative distributions of allele frequencies, which were markedly different when comparing recurrent and unstable v-SNPs (Fig. 3a, inset). We also found evidence of treatment shaping MTBC heterogeneity: the cumulative distributions progressively shift towards higher frequency alleles as treatment progresses (Additional file 1: Figure S8d).

The distribution of allele frequencies in MTBC implies that unstable, rare alleles are either constantly turned over or never able to expand beyond a certain point within the host. The first possibility would be consistent with a scenario where minor variants continuously bud from a predominant clone but are then selected against and therefore do not expand within the population (Fig. 3b). The second possibility would suggest that there is no predominant clone; instead, the overall MTBC population in the host is composed of a number of separate, but related, small populations (Fig. 3c), as has been proposed for *Burkholderia dolosa* colonization of cystic fibrosis patients [58] and more recently for untreated tuberculosis patients [56]. In this scenario, smaller populations would then be sampled sporadically, resulting in the overall appearance of instability. Studies of the dynamics of MTBC infection support both of these possibilities [14, 42, 50, 51, 55, 59]. An important distinction between the two scenarios is that the first relies on effective purifying selection to prune away minor variants, which in this context should bear a fitness cost; while in the second scenario the structured small populations would most likely be shaped by genetic drift and therefore less affected by the fitness of mutants. This distinction is relevant from the perspective of drug resistance: mutations conferring drug resistance often carry a fitness cost [60–63] and a predominance of purifying selection could therefore decrease the probability of resistant clones emerging during patient treatment, while the predominance of drift would not.

### Treatment efficacy impacts population dynamics

Since the generation of mutations is effectively random, while their impact on phenotype is not, deviation from the expected behavior of synonymous and nonsynonymous mutations can be used to infer underlying selection processes and distinguish genetic drift from purifying selection. We combined the ability to describe and analyze the temporal dynamics of recurrent v-SNPs with the fact that our patients received drug treatments of varying efficacy to address the impact of treatment on MTBC population dynamics.

**Fig. 4:**
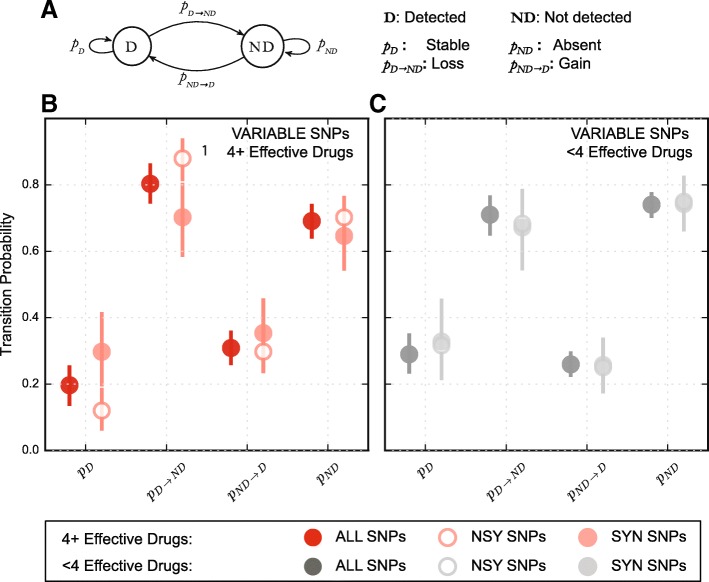
Allele dynamics in patients are congruent with purifying selection acting on MTBC populations treated with an efficacious drug combination. **a** We framed the allele dynamics within patients as a Markov process where alleles are either detected (*D*) or not detected (*ND*). We estimated each transition probability by re-sampling (N = 1000) the data with replacement. We stratified the SNPs by treatment efficacy experienced by the population and translational impact. The estimated transition probabilities for all alleles separated by translational impact showing the 95% confidence interval for **b** all v-SNPs in efficaciously treated patients (*red* symbols), **c** all v-SNPs in non-efficaciously treated patients (*dark gray* symbols). *NSY* nonsynonymous, *SYN* synonymous

To follow the fate of mutations during treatment, we considered the changes in population heterogeneity as a function of our ability to detect specific alleles over time. The presence and absence of a particular allele can be thought of as two distinct and exhaustive states. We thus set up a Markov chain to describe allele dynamics within the population and used allele frequency data from patients to estimate the relevant transition probabilities (Fig. 4a).

We observed that, as expected based on the frequency distributions of recurrent and unstable alleles, v-SNPs making up the majority of the genetic heterogeneity were also mostly transient in nature. Only 19.7% (13.5–25.7%, confidence interval (CI)^95%^) of v-SNPs in patients treated with four or more effective drugs and 29.0% (23.1%–35.3%, CI^95%^) of v-SNPs in patients treated with fewer than four effective drugs were detected at successive time points. We proceeded to estimate the transition probabilities for synonymous and nonsynonymous mutations separately. Synonymous v-SNPs were more likely to be stable than nonsynonymous v-SNPs in patients receiving four or more drugs: 29.7% (19.4–41.7%, CI^95%^) and 12.0% (6.0–18.9%, CI^95%^), respectively. There was also a slight indication that synonymous mutations may be more likely to emerge within the population of patients receiving four or more effective drugs (Fig. 4b). Neither of these observations were true for patients receiving fewer than four drugs, whose transition probability estimates showed no difference between synonymous and nonsynonymous mutations (Fig. 4c; Additional file 1: Table S5).

By definition, we cannot apply the same temporal dynamics analysis to unstable v-SNPs. However, we can determine whether there are any discrepancies between the observed and expected number of detected synonymous and nonsynonymous mutations present in the population. A tool commonly used to measure this is dN/dS [64]. In its canonical form, dN/dS is not suited to the analysis of genetic variation within evolving populations. This is because the measure of dN/dS relies on comparing mutations that are fixed within independently evolved populations (substitutions). Violating these assumptions compromises the ability to infer the strength of selection based on the absolute value of dN/dS. Nonetheless, the qualitative inference drawn from dN/dS in single populations mirrors that calculated for separate populations [65]. We therefore devised a measure based on these limitations, which we call the proportion of nonsynonymous to synonymous mutations (pNS). Conceptually, pNS provides a comparable insight as dN/dS; however, for its calculation we focus explicitly on codons that are mutated within a population when compared to a reference (polymorphisms). We then performed an in silico mutagenesis of affected codons to derive a null distribution of pNS under the scenario of neutral evolution. The comparison of pNS values calculated for our patients and those obtained by simulation allowed us to query the direction of selection.

**Fig. 5:**
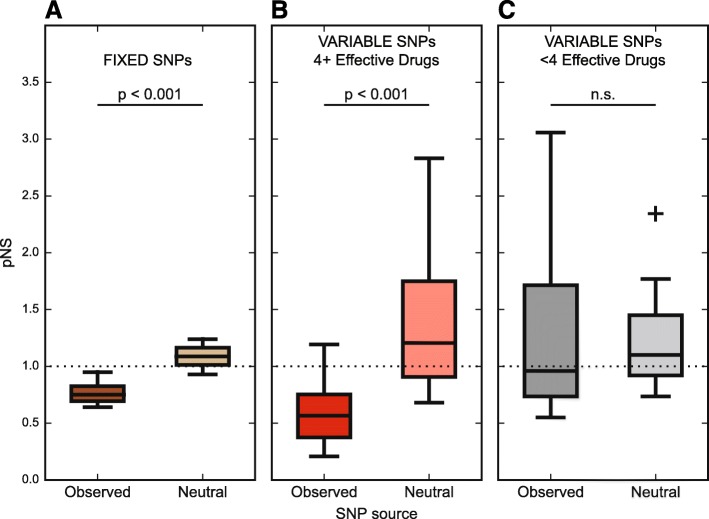
Efficacious treatment leads to a predominance of purifying selection of MTBC populations. **a** The proportion of nonsynonymous to synonymous mutations (*pNS*) for observed fixed SNPs in each patient (N = 12). We used computer simulation to estimate the outcome of mutating the same codons as were affected in patients but under a neutral scenario of genetic drift. **b** pNS calculated for each efficaciously treated patient at each time point (N = 30) with the corresponding neutral estimate. Patients given efficacious treatment show a pNS that is lower than expected in the absence of selection. **c** pNS calculated for each non-efficaciously treated patient at each time point (N = 21) with the corresponding neutral estimate. Patients given non-efficacious treatment do not show a significant decrease of pNS when compared to the expectation of no selection. All reported *p* values were calculated with the Mann Whitney U-test comparing the observed pNS to a simulated result generated using the assumption of genetic drift. *n.s.* not significant

We calculated the pNS for each sample using the observed SNPs. As a first step, we wanted to interrogate the pNS of f-SNPs across all patients. We found that it was significantly lower than expected under a scenario of neutral evolution (Mann–Whitney U-test, *p* = 2.35 × 10^−5^; Fig. 5a), thus supporting a role for purifying selection and recapitulating several reports in the literature [57, 66, 67]. When analyzing v-SNPs, we found that treatment had an impact on pNS. Specifically, patients treated with four or more drugs had a lower pNS than expected in the absence of selection (Mann–Whitney U-test, *p* = 1.10 × 10^−5^; Fig. 5b), while patients receiving fewer than four drugs did not (Mann–Whitney U-test, *p* = 0.29, Fig. 5c). We found no significant differences in pNS values calculated for time points across individual patients in a given category (Kruskal–Wallis H-test, *p* = 0.44 for patients receiving four or more drugs and *p* = 0.09 for patients receiving fewer than four drugs).

The pNS and Markov chain dynamics analyses provided congruous results pointing to an increased stability and possibly accumulation of synonymous mutations within MTBC populations in patients who received at least four effective drugs. In contrast, we observed no such trend in patients receiving fewer than four drugs. This disparity suggests that MTBC populations are subject to different selective forces depending on treatment efficacy. Furthermore, they point to purifying selection as an important mechanism shaping MTBC populations within patients receiving at least four effective drugs. This is a surprising finding, as antibiotic treatment is normally associated with positive selection of resistance determinants and compensatory mutations [31, 61, 68]. However, selective forces do not operate on the whole genome in a homogeneous manner [69]. It is therefore still possible that specific loci on the genome are under positive selection, but the signal is too weak to be detected on a genome-wide level. Analogous findings have been reported recently [55]. In light of these considerations, we explored the data for evidence of positive selection within our populations.

### Positive selection can occur with insufficient drug pressure

**Fig. 6:**
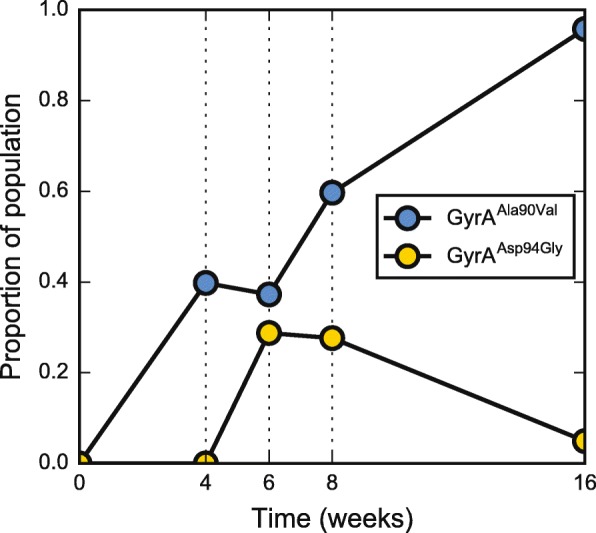
Emergence of fluoroquinolone resistance in patient 10 is driven by selection and modulated by clonal interference. The trajectory of estimated allele frequencies for two independent v-SNPs in gyrA: alanine 90 to valine (GyrA^Ala90Val^, *yellow dots*) and aspartate 94 to glycine (GyrA^Asp94Gly^, *blue dots*)

Extensive clinical evidence combined with multiple sequencing studies focusing on treatment failure provide ample examples of positive selection for antibiotic resistance traits from MTBC populations within a single patient [41–43, 55, 70]. Isolates from one of the patients in our study (patient 10) who received fewer than four effective drugs expanded their resistance spectrum in the course of treatment (Fig. 6). The gain of fluoroquinolone resistance manifested as the emergence of two separate populations of gyrase mutants. Four weeks after treatment began, approximately 40% of the reads from the patient supported a mutation in *gyrA* leading to an alanine to valine substitution at amino acid position 90 (A90V). While this clone eventually swept to fixation, it had to compete against a second clone carrying a substitution of aspartate to glycine at amino acid position 94 (D94G). Both of these mutations are known to lead to fluoroquinolone resistance [71]. We were able to discern the two as separate clones because two mutations (C to T at genomic position 1,284,167 and C to T at genomic position 3,767,194) shared temporal allele frequency trajectories with D94G but not A90V.

We were able to observe evidence of clonal expansion in 5 of our 12 patients (Additional file 1: Figure S9). In one case this expansion consisted entirely of synonymous mutations, in another a single mutation in an intergenic region swept across the population, while the remaining three cases included the expansion of nonsynonymous mutations (Additional file 1: Figure S10). Only MTBC in patient 10, described earlier, showed the expansion of clones carrying mutations known to confer an advantage in the face of drug pressure. Clonal expansion dynamics, however, are not necessarily evidence of positive selection and can occur readily in response to demographic changes (Additional file 1: Section 3) as well as genetic drift [72]. We therefore expanded our analysis to identify an excessive accumulation of mutations within segments of the genome that have previously been implicated in adaptation to drug pressure [38, 39, 55, 73].

We considered three gene sets relating to known adaptation to drug stress. These contained either bona fide drug resistance genes [73], genes associated with adaptation to drug resistance between patients [38, 39], or genes involved in mycolate biosynthesis that were reported to mediate adaptation to resistance within patients [55]. We also included a set of known T-cell antigens [74] under the assumption that the interplay between the pathogen and the host adaptive immune system may impose a positive selection pressure on the MTBC genome (see Additional file 5: Table S9 for a list of genes). We used a one-tailed binomial test to query whether there was an excess of mutations in the above gene sets compared to what would be expected given their size. We also assessed whether nonsynonymous mutations were over-represented in the accumulated v-SNPs compared to a schema of random mutation accumulation. We were unable to detect any evidence for excessive mutation in patients receiving four or more effective drugs (Table 1). By contrast, in patients treated with fewer than four effective drugs we identified an excess of mutations in validated drug target genes (binomial test *p* value 0.001), highlighting the existence of a drug pressure threshold that is necessary to suppress the emergence of resistance. If this threshold is not reached, as was the case in our patients receiving fewer than four drugs, the evolutionary constraint on the population is insufficient, allowing bacteria to acquire resistance.

**Table 1:** Excessive mutation of MTBC gene sets that are likely targets of positive selection

		4+ effective drugs		<4 effective drugs	
Gene set	N^a^	Excessive mutation^b^	Excess NSY^c^	Excessive mutation	Excess NSY
Drug resistance^d^	13	0/100 (0.501)	0/0 (1.000)	5/87 (0.001)	5/5 (0.177)
Drug resistance associated^e,f^	166	10/100 (0.545)	4/10 (0.987)	6/87 (0.946)	6/6 (0.121)
Mycolate superpathway^g^	54	3/100 (0.881)	1/3 (0.964)	5/87 (0.229)	3/5 (0.876)
MTBC T-cell antigens^h^	300	14/100 (0.153)	10/14 (0.426)	6/87 (0.550)	6/6 (0.121)

^a^Number of genes in the gene set

^b^Proportion of mutations in gene set, *p* value calculated with a one-sided binomial test

^c^Proportion of NSY mutations in gene set, *p* value calculated with a one-sided binomial test

^d^[73]

^e^[38]

^f^[39]

^g^[55]

^h^[74]

## Discussion

We combined very deep DNA sequencing with serial sputum sampling to gain a detailed view of MTBC population structure and dynamics within patients undergoing treatment. The first feature to emerge from our data was the wealth of genetic heterogeneity that is present in the population. Strikingly, the major contributors to genetic diversity were very rare variants that were detected only once across multiple sputum samples obtained from individual patients. There are several possible explanations for this result. The most trivial stems from the sequencing depth and the analytical challenge of distinguishing true minor variants from sequencing errors. While we did not systematically confirm individual v-SNP calls, an important limitation to our analyses, we deliberately chose a combination of stringent criteria to limit the impact of false positive v-SNP calls. Our approach mirrored previously published strategies [50, 56], and we excluded a large number of low-frequency v-SNPs from further analysis (Additional file 1: Figure S4). However, each sampling included an in vitro expansion of the MTBC population during bacterial culture prior to sequencing; we therefore cannot completely exclude the possibility that part of the observed variation arose during the culturing step. Nonetheless, we posit this is unlikely, as the number of v-SNPs varied over time and across patients. Moreover, we observe significantly more v-SNPs at each time point in patients treated with fewer than four drugs (Additional file 1: Figure S7), a fact that reflects probable biological differences among patients, making a consistent technical bias improbable. An alternative explanation for the high abundance of transient minor variants is imperfect sampling. It is possible that the populations sampled through sputum do not fully reflect the true heterogeneity of MTBC populations in the lung. This possibility illustrates the variable probability of granulomas gaining access to airways and might not provide any further indication of the selective forces shaping the MTBC population. We describe evidence for the stochastic nature of sampling in our data as the incongruence between parallel re-samplings of the same patient (Fig. 2). In a recent report of a detailed sampling of MTBC populations across several foci of infection within deceased TB patients, Lieberman et al. [56] showed a large genetic diversity within each patient that may not be completely represented in tracheal aspirates—a proxy the authors used for sputum samples. Furthermore, several studies found a comparable degree of genetic diversity of MTBC within and between hosts [42, 54, 59]; these observations complement radiological findings of granuloma dynamics [14]. In the majority of these studies, authors comment on the transience of the detected polymorphisms and highlight the importance of sampling the host population as fully as possible. The latter is crucial for drawing conclusions regarding transmission chains and determining resistance profiles based on nucleic acid amplification techniques. Our findings complement these views and emphasize the fact that sputum samples are not uniform or equivalent. In addition they highlight a need for further studies aimed at improving on diagnostic algorithms, to ensure sufficient sampling in subpopulations of patients that may return erroneous results. Examples of such subgroups include patients with prior history of TB, patients with compliance issues, and patients that are likely to have a mixed infection. An upshot of the limited compositional congruence across sputum samples is that sufficiently frequent sampling should reflect the population to a suitable degree. Specifically, the fact that 84.4% (314/374 polymorphic sites) of the minor alleles are only ever detected in a single sputum sample suggests that there may be something beyond stochastic sampling that could account for allele instability within our patients. One possibility is that bacteria giving rise to transient heterogeneity represent a specific subset of resolving granulomas. An alternative possibility is that the transience of many alleles has a biological cause and it reflects a dynamic exploration of the mutational space constrained by selection.

Having grouped the patients based on the number of effective drugs they received, we were able to leverage the temporal dynamics of minor alleles to investigate the selective forces that shape MTBC diversity. While the fact that the pNS metric has not been extensively characterized calls for a cautious interpretation of our results, we conclude that purifying selection is the main force shaping MTBC populations within patients receiving at least four effective drugs. The combined effect of multiple drugs seems to ensure that resistance to any individual drug poses too much of a fitness disadvantage to be retained within the population. An important consequence of the virtual absence of horizontal gene transfer in MTBC is that all loci are linked; as a result, variants providing only a slight advantage are more likely to be lost from the population since the benefit they provide is probably less than the cost of any linked deleterious mutation. This process is termed background selection [75, 76] and may be the underlying mechanism suppressing the signal for positive selection in our study and MTBC populations at large [57]. These considerations no longer hold in patients receiving fewer than four effective drugs, where the combined effect of drugs is inadequate and does not impose a sufficient fitness cost to the emergence of resistance. Our conclusions add an important piece to the understanding of the interplay between host immunity and antibiotics in controlling MTBC infection. Lieberman and colleagues [56] reported that, in the absence of antibiotic treatment, MTBC within patients is likely to be constrained by purifying selection, while positive selection of antibiotic resistance traits is prevalent in single patients failing treatment [41–43, 70]. The question arises: why does the immune system not constrain the emergence of resistance? Our findings indicate that the fitness landscape of MTBC populations treated with insufficient drugs allows for resistance and infectiousness to co-exist. Imposing sufficient drug stress is therefore necessary to close off those evolutionary trajectories.

The main drivers of the global genetic diversity of MTBC are believed to be the recent demographic expansion of the human population combined with genetic drift and purifying selection [57, 66]. These features speak of a pathogen that is well adapted to the human host [77]. It is therefore important to highlight that the dynamics we report for patients receiving at least four effective drugs reflect this high degree of adaptation. They are markedly different from those observed during chronic infection of cystic fibrosis patients with opportunistic *B. dolosa* [58, 78] or *Pseudomonas aeruginosa* [79, 80]. Opportunists need to undergo significant adaptation to the new host environment. This is manifested by clear indication of positive selection through convergent evolution of traits necessary to establish and maintain infection—pathoadaptive mutations. The dynamics of MTBC differ also from those of other human-adapted bacteria. The analysis of within-patient evolution of *Helicobacter pylori* and *Staphylococcus aureus* both speak of an ongoing purifying selection, but with a component of diversifying selection, where the continued carriage of these bacteria within the human host is dependent upon their continuous immune escape [81, 82]. We found no evidence of diversifying selection acting on known T-cell antigens of MTBC, reinforcing the view that these antigens are under purifying selection, reflecting the strategy MTBC uses to subvert host immunity [67, 74, 83].

## Conclusions

Combination therapy in TB is effective at suppressing drug resistance by restricting the evolutionary options through purifying selection. However, unlike the accepted view that MTBC is genetically stable [84], we find evidence of considerable and perhaps continuous turnover of genetic variants within hosts. As a consequence, the erosion of drug pressure, through the administration of a suboptimal treatment regimen, allows the emergence of drug-resistant clones. Importantly, these clones may already be present in the constantly generated genetic diversity of the MTBC populations within the host. This possibility explains why it is often the case that multiple clones carrying resistance to the same drug arise virtually simultaneously within a patient—we report the emergence of two fluoroquinolone-resistant clones within patient 10 (Fig. 6), and similar examples have been reported by many studies [41–43].

The importance of timely drug susceptibility testing and appropriate regimen composition is well established. We would like to put forward the suggestion that, in some cases, susceptibility testing should be done on multiple parallel sputum samples. Our observations regarding the heterogeneity of the composition of sputum samples clearly shows that even three samples obtained within 24 h from each other are not uniform and may result in different resistance profiles. Such information should lead to better treatment and lower probability of resistance expansion. Finally, our findings have clear implications for future clinical trials designed to test the efficacy of novel treatment regimens. We propose that monitoring population dynamics within patients during trials would provide an informative metric for assessing regimen efficacy. Specifically, effective regimens should carry the signature of purifying selection.

## Methods

### Study cohort and sample collection

The study to investigate the range of tuberculosis presentation and treatment (NCT01071603, clincaltrials.gov) conducted in Henan Provincial Chest Hospital (HPCH) was approved by the HPCH and National Institute of Allergy and Infectious Diseases institutional review boards. The methods of this study were carried out in accordance with the approved guidelines and written informed consent was obtained from the subjects prior to the study. During this study, 52 smear-positive TB patients were enrolled. For these patients, time-serial isolates were collected at seven time points: at enrollment (before treatment) and 2, 4, 6, 8, 16, and 24 weeks after treatment. At each time point for each patient, three sputum samples were collected (night sputum, morning sputum, immediate sputum). As treatment progressed, some patients became both smear- and culture-negative. As a result the number of collected sputum samples varied. Thus, from the 52 patients, we selected 12 that presented more serial isolates and were more representative regarding differential drug susceptibilities/treatment efficacies. We focused specifically on the first 8 weeks of treatment. The isolates of the 12 patients are described in Fig. 1. Among the 12 patients, five were drug-sensitive (patients 1–5), three were INH-resistant (patients 6–8), two were MDR (patients 9 and10). None of these ten patients had prior history of TB. Two patients had MDR-TB and had been treated before for active TB (patients 11 and 12). Patients 1–8 received four or more effective drugs while patients 9–12 received fewer than four effective drugs.

### Isolate culture and deep whole genome sequencing

Each sputum sample was decontaminated and inoculated onto both solid Löwenstein–Jensen (L-J) and liquid mycobacterium growth indicator tube (MGIT) broth. This resulted in three L-J cultures and three MGIT cultures at each time point. Cultures from the early weeks of treatment often had hundreds of colonies on the L-J medium. We scraped the colonies from the slope surface and extracted DNA from the population using the CTAB method as described before [42] for deep population sequencing. As treatment progressed and the bacterial load in the patient decreased, the number of colonies on L-J medium decreased. In these cases we extracted DNA from whole MGIT cultures to represent the population of the bacteria in the patient. Overall, 39 isolates were sequenced from L-J medium and 14 isolates from MGIT medium (Additional file 1: Table S3). Whole genome sequencing was performed on an Illumina HiSeq 2000 instrument and the average sequencing depth was ~1000-fold for each isolate. We generated between 6–10 GB of sequencing data for each sample.

### Empirical sequencing error estimation

We aimed to evaluate the impact of PCR and sequencing errors as well as the emergence of minor variants during the in vitro expansion of bacterial colonies. We used a two-pronged approach. On the one hand, we picked two single colonies from L-J medium and expanded them in vitro. These colonies came from sputa that were not related to those used for the rest of the study. We extracted the DNA of each single colony as described above and prepared two DNA libraries for each single colony, giving us a total of four samples. Each library was sequenced on an Illumina HiSeq 2000 platform with the same strategy as above. On the other hand we used ART [85] to simulate synthetic next-generation sequencing reads using the genome of *M. tuberculosis* CCDC5079 (GenBank CP001641) as a template. We simulated reads using our own read error model and quality profiles with parameters set according to our sequencing platform and strategy. A total of 500 synthetic paired-end sequencing files were simulated, with an average depth of 850.

### Identification of fixed and variable mutations

The pipeline we used for fixed mutations and unfixed mutations obtained from all our sequencing runs and read simulations was published before [42]. Briefly, scythe (https://github.com/ucdavis-bioinformatics/scythe) and sickle (https://github.com/ucdavis-bioinformatics/sickle) were used for read trimming, bwa [86] was used for mapping, and SAMtools [87] was used for SNP calling. Two reference genomes were used as template: one was the standard reference strain, *M. tuberculosis* H37Rv (GenBank AL123456) [88], and the other was CCDC5079 (GenBank CP001641) [89], a Beijing isolate genetically close to our strains (see Additional file 1: Figure S11 for more phylogenetic details). We used LoFreq [90] and VarScan 2 [91] to call intra-host variable mutations (v-SNPs) and considered only congruous calls. We further defined a set of thresholds to exclude sequencing errors and false positives. v-SNP calls were only made if the mapping quality of the read was above 30 and the Phred score for base quality exceeded 20. Further, we required that each v-SNP was supported by at least five reads, with no fewer than two reads for each sequencing direction. We discarded v-SNPs for which we detected evidence for strand bias in the reads supporting it. Next we excluded v-SNPs for which support was significantly enriched (Kolmogorov–Smirnov test) in the terminal parts of the read. We ignored all SNPs that arose in repetitive regions of the genome (e.g., PPE/PE-PGRS genes). We manually removed v-SNPs that showed patterns consistent with sequencing noise (e.g., occurrence of the same minor variant substitution in several patients, spurious proximal mutations). Finally, we considered only v-SNPs whose frequencies were estimated to be ≥1.5% in at least one sampled population.

### Data analysis and code availability

We performed all data manipulation and analyses using custom scripts written in Python, including specialized packages NumPy, SciPy [92], pandas [93], and scikit-learn [94], and interfaced with iPython [95]. We generated the majority of our figures using the Python matplotlib [96] package. We performed mixed model linear regression analyses using the Python package scikit-statsmodels [97]. Statistical analyses were based, where appropriate, on non-parametric methods. Mann–Whitney U-test was used for distribution comparison of pNS. 95% confidence intervals were derived empirically using re-sampling techniques. Excess mutation of genetic regions was examined using Fisher’s exact test, paired with a downstream one-sided binomial test to establish the likelihood of the observed outcome.

With the exception of sequencing data (see accession numbers below), we deposited all the data into a public repository (doi:10.5281/zenodo.322377) and made all the analysis scripts available through a public repository (doi:10.5281/zenodo.345135) and at GitHub (https://github.com/swisstph/TBRU_serialTB).

### Site frequency spectrum analysis

We estimated allele frequencies by computing the proportion of reads supporting a mutant allele within the total number of reads that mapped to a given region, provided they fulfilled the quality criteria outlined in “Identification of fixed and variable mutations”. We obtained estimates of allele frequencies using LoFreq, as outlined in “Identification of fixed and variable mutations”. We produced the folded site frequency spectrum by plotting a histogram of all the estimated allele frequencies for v-SNPs. SNPs that were fixed within the population at the point of diagnosis were not included.

### Markov chain analysis of allele dynamics

We explored the temporal dynamics of individual alleles found in our samples by assuming that the stability of alleles is random. This allowed us to describe the presence (“Detected”) or absence (“Not Detected”) of alleles at a given time as two mutually exclusive and exhaustive states that define a Markov chain (Fig. 4a). We grouped all the v-SNPs based on treatment efficacy and used their presence or absence at each time point as the basis for the estimation of transition probabilities. We define four different transition probabilities: Detected—Detected as “Stable”, Detected—Not Detected as “Loss”, Not Detected—Detected as “Gain”, and Not Detected—Not Detected as “Absent”. The following pairs of transition probabilities sum to 1.0 by definition: Stable–Loss and Absent–Gain.

We used custom Python scripts to randomly re-sample with replacement (bootstrap) the v-SNPs and in each case count the number of occurrences of each type of transition. We then calculated what proportion of the possible outcomes each type represented and thus obtained an estimate of transition probabilities for each state. We performed 1000 iterations of the bootstrap, sorted the estimates and took the 50th percentile value as the estimate of the probability. We defined the 95% confidence interval (CI^95%^) by taking the 25th and 975th permille values of the sorted estimates. We performed the same analysis on SNPs separated by translational impact (nonsynonymous and synonymous). In addition we repeated the analysis for all SNPs and synonymous and nonsynonymous SNPs using only v-SNPs whose estimated frequency exceeded 1.5%.

### pNS analysis

We calculated the proportion of nonsynonymous to synonymous mutations by first annotating the detected SNPs by using either Var Scan2 (v-SNPs) or custom Python scripts (f-SNPs). We omitted all SNPs that did not affect a coding sequence.

We then generated a codon substitution matrix using a base substitution model that takes into account the proportion of guanine and cytosine in the genome (percentage GC content, 0.656) and the proportion of transitions that occurred at the final position of codons in synonymous f-SNPs (Ti, 0.729) as described previously [98]. Briefly, for each codon we used a custom Python script to simulate 50,000 individual introductions of a single mutation into the codon, and scored the outcomes as either synonymous or nonsynonymous. We considered the average number of nonsynonymous outcomes of the simulations as an estimate of the probability that a mutation in the given codon would be nonsynonymous.

We used the following formula to calculate the pNS for each sample:

$$ p N S=\frac{\frac{{\mathrm{NSY}}_{\mathrm{observed}}}{{\displaystyle \sum_{i=1}^n \Pr {(NSY)}_i}}}{\frac{{\mathrm{SYN}}_{\mathrm{observed}}}{{\displaystyle \sum_{i=1}^n \Pr {(SYN)}_i}}} $$

where NSY_observed_ represents the number of observed nonsynonymous and SYN_observed_ represents the number of observed synonymous mutations in the sample. Pr(NSY)_*i*_ represents the expected probability of a nonsynonymous mutation arising from the *i*-th codon, which is the same as the *i*-th codon mutated in the observed samples. *n* represents the number of all the mutated codons in the sample.

In order to be able to test the hypothesis that the observed pNS shows a deviation from the null expectation of genetic drift, we simulated the outcome of mutating the same codons under the assumption of random mutagenesis. To this effect we consider the mutation of each codon that was mutated within a sample as a Bernoulli trial with the probability of success given by the expected probability of nonsynonymous mutation calculated earlier. We also assumed that mutations occurred independently of each other. Performing a Bernoulli trial for each mutated codon in the sample generated a dataset that reflected a possible distribution of outcomes in the absence of selection. We used this outcome as the source of “observed” variables for the calculation of pNS in the formula above.

We grouped the pNS calculations for our samples and their cognate simulations based on treatment efficacy and performed a Mann–Whitney U-test to determine whether the two sets of pNS results could come from the same population. Finally, we repeated the pNS analysis, simulation, and U-test considering only v-SNPs whose estimated frequency exceeded 1.5%.

### Excess mutation accumulation

We focused the excess mutation accumulation analysis exclusively on v-SNPs accumulated after the onset of treatment, therefore ignoring all mutations that were present before treatment began. We considered a given gene set to be excessively mutated if it had accumulated more mutations than we would expect by chance given its nucleotide length. The null expectation is based on the binomial distribution where the probability of success is given as the proportion of the genome covered by the gene set of interest, the number of trials is the total number of mutations detected across the tested samples, and the number of successful trials is the number of mutations that affected the gene set of interest. We split our patients into two groups based on the number of effective drugs in their regimen and then performed a one-tailed binomial test for genes covering different gene sets (see Additional file 5: Table S9 for gene lists).

We determined whether or not there was an excessive accumulation of nonsynonymous mutations within the mutations affecting each gene set by using a similar approach, excluding all mutations that were present at the onset of treatment. The binomial distribution was defined as follows: the probability of success was given by the expected probability of a nonsynonymous mutation given the mutated codons affecting a gene set of interest (as described in “pNS analysis”); the number of trials was defined as the total number of intragenic mutations within a gene set of interest; and the number of successful trials as the number of nonsynonymous mutations within the same set. We carried out these determinations for both groups of patients and then performed a one-tailed binomial test for genes covering different gene sets.

### Accession codes

Sequencing reads have been submitted to the EMBL-EBI European Nucleotide Archive (ENA) Sequence Read Archive (SRA) under the study accession number PRJEB13325 and PRJEB17864.

## Additional files

Additional file 1: Supplementary text, figures and tables. In this document we elaborate on the efficacy of treatment (Section 1), expand on the reasoning behind our choice of variant calling approach, describe the outcome of population simulations providing the basis for our demographic expectations, illustrate the phylogeny of our isolates, and finally describe the data contained in the other additional files. The display items in the document include the following tables and figures. **Table S1** Phenotypic drug susceptibility testing for first line drugs. **Table S2** Treatment combinations given to patients. **Table S3** Average sequencing depth of individual samples. **Table S4** Variations in the number of detected f-SNPs in patients at each time point is consistent with v-SNP dynamics. **Table S5** Transition probabilities for the detection of v-SNPs. **Figure S1** Treatment efficacy in patient groups. **Figure S2** Error profiles of simulated reads. **Figure S3** Culture-induced minor variants in MTBC. **Figure S4** Retained minor variants in our patient population. **Figure S5** Demographic trends of MTBC populations within the host are consistent with bacterial killing. **Figure S6** Sequencing depth does not bias v-SNP detection. **Figure S7** The number of detected v-SNPs for each patient varies over time. **Figure S8** Cumulative distributions of allele frequencies. **Figure S9** Frequency trajectories of recurrent alleles vary, showing a dynamic and heterogeneous population. **Figure S10** Sweeps of v-SNPs occur independently of the type of v-SNP affected the association of v-SNPs with drug resistance. **Figure S11** Most patients are infected with strains of the Beijing family. (PDF 532 kb)

Additional file 2: Patient-level data on bacterial populations, including the heterogeneity of the population and the burden of infection. (CSV 1 kb)

Additional file 3: v-SNP data. All collected and calculated information for the detected v-SNPs. (CSV 36 kb)

Additional file 4: f-SNP data. All collected and calculated information for the detected f-SNPs. (CSV 186 kb)

Additional file 5: Published predefined gene sets. These gene sets were used for mutation enrichment analysis (Table 1 in the main text). (CSV 41 kb)
