# Supplementary material for: The within-host population dynamics of Mycobacterium tuberculosis vary with treatment efficacy
Source: Genome Biol. 2017 Apr 19;18:71. doi: 10.1186/s13059-017-1196-0 (PMC5395877; doi:10.1186/s13059-017-1196-0)
Supplement: Supplementary file 1 — Supplementary text, figures and tables. In this document we elaborate on the efficacy of treatment (Section 1), expand on the reasoning behind our choice of variant calling approach, describe the outcome of population simulations providing the basis for our demographic expectations, illustrate the phylogeny of our isolates, and finally describe the data contained in the other additional files. The display items in the document include the following tables and figures. Table S1 Phenotypic drug susceptibility testing for first line drugs. Table S2 Treatment combinations given to patients. Table S3 Average sequencing depth of individual samples. Table S4 Variations in the number of detected f-SNPs in patients at each time point is consistent with v-SNP dynamics. Table S5 Transition probabilities for the detection of v-SNPs. Figure S1 Treatment efficacy in patient groups. Figure S2 Error profiles of simulated reads. Figure S3 Culture-induced minor variants in MTBC. Figure S4 Retained minor variants in our patient population. Figure S5 Demographic trends of MTBC populations within the host are consistent with bacterial killing. Figure S6 Sequencing depth does not bias v-SNP detection. Figure S7 The number of detected v-SNPs for each patient varies over time. Figure S8 Cumulative distributions of allele frequencies. Figure S9 Frequency trajectories of recurrent alleles vary, showing a dynamic and heterogeneous population. Figure S10 Sweeps of v-SNPs occur independently of the type of v-SNP affected the association of v-SNPs with drug resistance. Figure S11 Most patients are infected with strains of the Beijing family. (PDF 532 kb) [file 13059_2017_1196_MOESM1_ESM.pdf]

The within-host population dynamics of *Mycobacterium tuberculosis* vary with treatment efficacy.

Supplementary Material

Andrej Trauner[1,2,a], Qingyun Liu[3,7,a], Laura E. Via[4,5], Xin Liu[6], Xianglin Ruan[6], Lili Liang[6], Huimin Shi[6], Ying Chen[6], Ziling Wang[6], Ruixia Liang[6], Wei Zhang[6], Wang Wei[6], Jingcai Gao[7], Gang Sun[3,7], Daniela Brites[1,2], Kathleen England[4], Guolong Zhang*[7,8], Sebastien Gagneux†[1,2], Clifton E. Barry III‡[4,5], and Qian Gao§[3,7]

[1]Department of Medical Parasitology and Infection Biology, Swiss Tropical and Public Health Institute, 4001 Basel, Switzerland

[2]University of Basel, 4001 Basel, Switzerland

[3]Key Laboratory of Medical Molecular Virology of Ministries of Education and Health, Institutes of Biomedical Sciences and Institute of Medical Microbiology, School of Basic Medical Sciences, Fudan University, Shanghai 200032, China

[4]Tuberculosis Research Section, Laboratory of Clinical Infectious Diseases, NIAID, NIH, Bethesda, MD, 20892, USA

[5]Institute of Infectious Disease and Molecular Medicine, and the Department of Clinical Laboratory Sciences, Faculty of Health Sciences, University of Cape Town, Rondebosch 7701, Republic of South Africa

[6]Henan Provincial Chest Hospital, Zhengzhou 450003, Henan, China

[7]Sino-US International Research Centers of Tuberculosis, Zhengzhou 450003, Henan, China

[8]Henan Public Health Clinical Center, Zhengzhou 450003, Henan, China

[a]These authors contributed equally.

*guolongzhang@hotmail.com

†sebastien.gagneux@unibas.ch

‡cbarry@niaid.nih.gov

§qgao99@yahoo.com

List of Tables

S1 **Phenotypic drug susceptibility testing for first line drugs.** . 3
S2 **Treatment combinations given to patients.** 4
S3 **Average sequencing depth of individual samples.** 5
S4 **Variations in the number of detected f-SNPs in patients at each time point is consistent with v-SNP dynamics.** 6
S5 **Transition probabilities for the detection of v-SNPs** 7

Contents

1 Effect of treatment **8**

2 Variant calling **9**
2.1 Automatic filtering 9
2.2 Empirical error filtering 9
2.3 Culture-induced variation 11

3 Demographic indicators of bacterial killing **15**

4 Phylogenetic analysis of patient isolates **23**
4.1 Methodology 23
4.2 Majority of study isolates belong to the Beijing family 23

5 Supplementary datasets **25**
5.1 Patient data 25
5.2 v-SNP data 26
5.3 f-SNP data 27
5.4 Predefined Genesets 27

Table S1: In all cases the results are given for Isoniazid-Rifampicin-Ethambutol-Streptomycin. **S**: susceptible, **R**: resistant. **L-J**: Löwenstein-Jenssen medium, **MGIT**: Mycobacterial growth indicator tube.

	Week 0		Week 16	Week 24
Patient ID	L-J	MGIT	MGIT	MGIT
Patient01	S-S-S-S	S-S-S-S		
Patient02	S-S-S-S	S-S-S-S		
Patient03	S-S-S-S	S-S-S-S		
Patient04	S-S-S-S	S-S-S-S		
Patient05	S-S-S-S	S-S-S-S		
Patient06	R-S-S-S	R-S-S-S		
Patient07	R-S-S-R	R-S-S-R		
Patient08	R-S-S-S	R-S-S-S		
Patient09	R-R-S-R	R-R-S-R	R-R-S-R	R-R-S-R
Patient10	R-R-R-R	R-R-R-R	R-R-R-R	R-R-R-R
Patient11	R-R-S-R	R-R-S-R		
Patient12	R-R-S-R	R-R-S-R	R-R-S-R	

Table S2: Arrows (→) indicate the continuation of the treatment from the previous week. **A**: amikacin, **B**: rifabutin, **C**: clofazimine, **E**: ethambutol, **G**: gatifloxacin, **H**: isoniazid, **L**: levofloxacin, **M**: moxifloxacin, **P**: PAS, **Q**: rifapentin, **R**: rifampicin, **T**: prothionamide **Z**: pyrazinamide

	Week						
Patient ID	0	2	4	6	8	16	24
Patient01	HRZE/LP	→	→	RZE/L	E/A	E/Q	HE/LQP
Patient02	HRZE/L	→	→	→	→	HRE	→
Patient03	HRZE/LP	→	→	→	→	→	HRZE
Patient04	HRZE/LP	→	→	→	→	RZE/LP	→
Patient05	HRZE/LP	→	Interruption	HRZE/LP	HRZE/LM	→	→
Patient06	HRE/L	HRZE/L	→	→	→	→	→
Patient07	HRZE/L	→	HRZE/P	HRZE/LP	→	HZE/LPQ	HZ/LPQ
Patient08	HRZE/L	HRZE/LP	→	→	→	HZE/LPQT	→
Patient09	HRZE/LPA	→	→	→	HRZE/L	→	→
Patient10	HRE/L	→	HRZE/L	HZE/LPB	→	HZE/LPBT	→
Patient11	HRZE/LPA	→	→	→	RZE/LPAB	HE/LPABGT	→
Patient12	R/G	HE/BGP	HE/BGCP	HE/AGCP	→	→	Died

Table S3: The values represent averages of coverage across the genome. The DNA used for sequencing was extracted from cultures grown on either Löwenstein-Jenssen medium (no emphasis) or from Mycobacterial Growth Indicator Tubes (MGIT, bold text).

	Week				
Patient ID	0	2	4	6	8
Patient01	1106.8	1482.9	1266.7	**1515.6**	**2552.0**
Patient02	1424.5	**1360.2**	**1269.4**	1454.0	
Patient03	1311.7	1039.1		708.8	**1284.8**
Patient04	948.0		**1097.8**		
Patient05	1026.5	**1059.2**	1018.3	**956.4**	**1562.6**
Patient06	1388.3	353.4	1431.0	1339.4	**1308.3**
Patient07	1398.2	1093.7	1683.1	855.1	**991.8**
Patient08	775.9	1350.7	1425.0	1356.9	1002.2
Patient09	1237.1	858.0	1532.2	1718.9	
Patient10	2101.0		1262.8	940.8	1372.6
Patient11	1664.6	700.2	**1485.8**	**1431.6**	**1268.8**
Patient12	1724.7	904.3	822.6	1790.7	1422.4

Table S4: Numbers in bold indicate increases in detected f-SNPs, while those in italics represent a decrease in f-SNPs. All gains of f-SNPs can be attributed to sweeps within populations - see **Figure S9**

		Week				
Patient ID	Reference	0	2	4	6	8
Patient01	CCDC5079	162	162	162	162	162
Patient02	CCDC5079	200	200	200	**202**	
Patient03	CCDC5079	202	202		202	202
Patient04	CCDC5079	369		369		
Patient05	CCDC5079	221	221	221	221	221
Patient06	CCDC5079	1137	1137	1137	1137	1137
Patient07	CCDC5079	216	216	216	**217**	216
Patient08	CCDC5079	200	200	200	200	200
Patient09	CCDC5079	195	195	195	*194*	
Patient10	CCDC5079	203		**205**	**205**	203
Patient11	CCDC5079	563	563	563	*552*	563
Patient12	CCDC5079	209	209	209	209	209

Table S5: Calculations performed by re-sampling data as described in the Online methods. The p_D transition: stable allele, $p_D \rightarrow p_{ND}$: loss of allele, p_{ND}: absence of an allele, $p_{ND} \rightarrow p_D$: gain of an allele.

		p_D		$p_D \rightarrow p_{ND}$		p_{ND}		$p_{ND} \rightarrow p_D$	
Treatment	SNPs	mean	$CI^{95\%}$	mean	$CI^{95\%}$	mean	$CI^{95\%}$	mean	$CI^{95\%}$
4+ Drugs	All	0.197	0.135-0.257	0.803	0.743-0.865	0.691	0.639-0.743	0.309	0.257-0.361
	Nonsynonymous	0.120	0.060-0.189	0.880	0.811-0.940	0.702	0.639-0.767	0.298	0.233-0.361
	Synonymous	0.297	0.194-0.417	0.703	0.583-0.806	0.646	0.542-0.750	0.354	0.250-0.458
<4 Drugs	All	0.290	0.231-0.353	0.710	0.647-0.769	0.741	0.701-0.778	0.259	0.222-0.299
	Nonsynonymous	0.317	0.244-0.396	0.683	0.604-0.756	0.750	0.699-0.802	0.250	0.198-0.301
	Synonymous	0.328	0.212-0.458	0.672	0.542-0.788	0.742	0.660-0.828	0.258	0.172-0.340

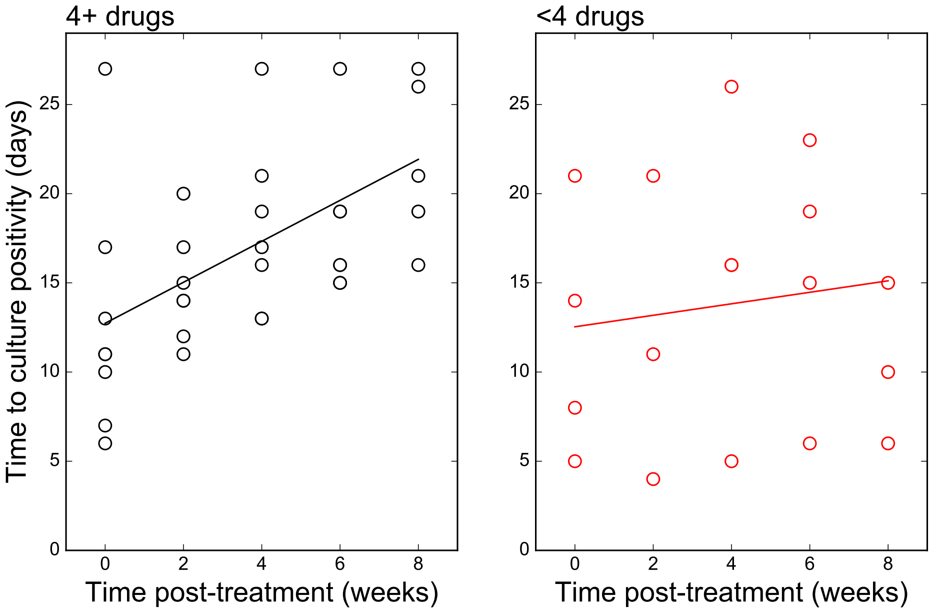

Figure S1: Treatment efficacy in patient groups.

1 Effect of treatment

Antibiotic treatment results in the decrease of bacterial populations within patients. Sputa from our patients were routinely used to inoculate Mycobacterial Growth Indicator Tubes (MGIT). The protocol for inoculation requires a standardisation of bacterial input based on optical density. As a result we used changes in time to culture positivity as a proxy for the decrease in the size of the bacterial population within the host. We used Mixed model linear regression to quantify the impact of treatment efficacy on bacterial burden. We saw clear differences in the treatment composition, with patients receiving at least four effective drugs (4+ drugs) reducing their bacterial burdens more effectively; see **Figure S1**.

Regression parameters for time to culture positivity

	coefficient	Std.Err.	z	P>|z|	[0.025	0.975]
Intercept	12.736	1.882	6.766	0.000	9.046	16.425
Slope	1.148	0.247	4.649	0.000	0.664	1.633
ΔIntercept (<4 DRUGS)	-0.198	3.271	-0.061	0.952	-6.609	6.213
ΔSlope (<4 DRUGS)	-0.826	0.417	-1.983	0.047	-1.643	-0.010

2 Variant calling

2.1 Automatic filtering

The methodology for the determination of minor variants within populations is currently being actively developed by a number of different groups with new tools emerging on a regular basis. We have used an approach based on the strategy used by Lieberman *et al.* (1) by first filtering the variant calls such that they fulfil the following criteria:

- Mapping quality 30+ and base quality 20+ to decrease sequencing artefacts,
- A coverage of at least two reads in each sequencing direction to decrease strand bias with a total minimum coverage for the minor variant of 5-fold,
- An average tail distance of between 9 and 49 bases to minimise tail effects,
- Removal of genes with repetitive regions (e.g. PE/PGRS genes, insertion elements...), as defined by Coscolla *et al.* (2),

Next, we performed minor variant calls with VarScan 2 (3) and LoFreq (4). We kept only variants whose call was congruous across the different methodologies.

2.2 Empirical error filtering

There are two major sources of systematic noise affecting our samples:

1. Technological limitations related to sequencing: both PCR and the sequencing platform have an inherent error profile.
2. Each sputum sample was cultured *in vitro* to expand the *M. tuberculosis* population prior to DNA extraction. This expansion may have introduced minor variants into the population that were not present in the host.

We started by addressing the techincal limitations first. Within our analytical pipeline we already account for poor base and mapping quality, and remove data that do not fit our criteria. The issue we still had to address is the identification of spurious variants that carry the profile of legitimate variants. To this end we used ART (5) to generate synthetic reads carrying the error profile of our sequencing data (derived with the "*art_profiler_illumina*" toolkit). We used the genome of *M. tuberculosis* strain H37Rv (GenBank: AL123456) as the template to simulate 500 sequencing runs with an average genome coverage of 1000-fold. Our expectation was that any minor variants to occur from this approach would be purely due to

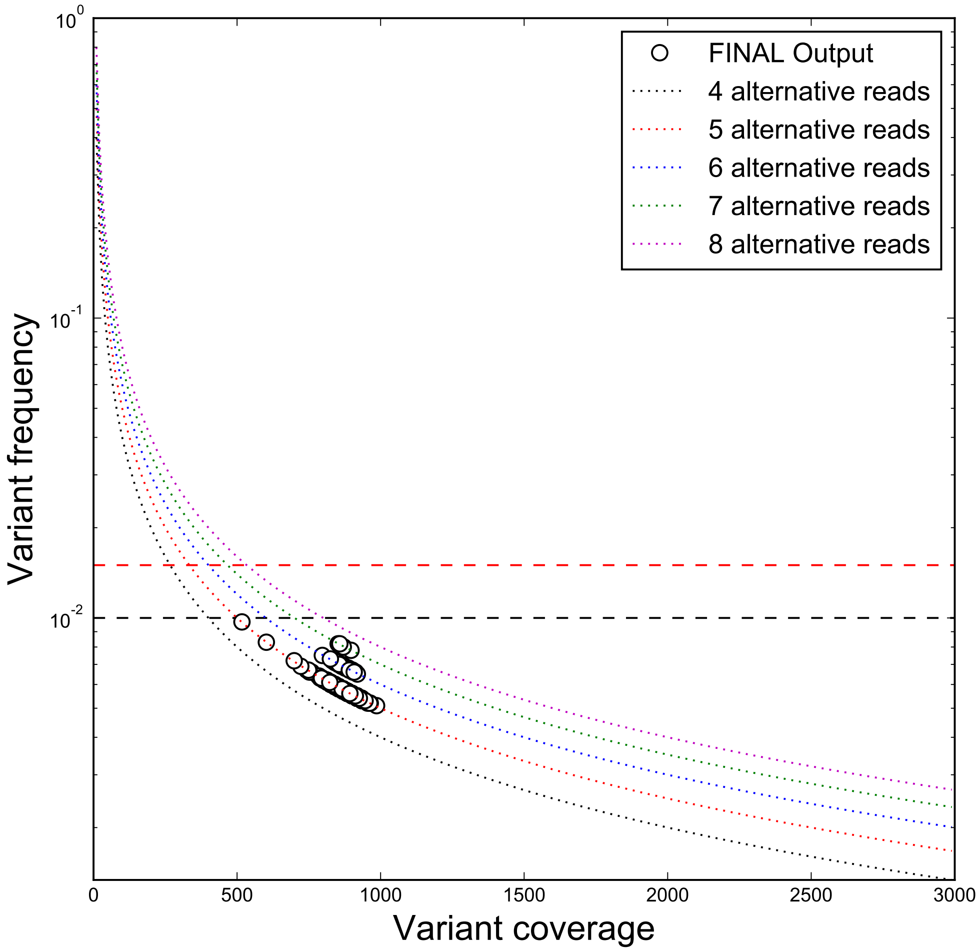

Figure S2: Error profiles of simulated reads. We simulated 500 sequencing runs with a target average genome-wide coverage of 1000-fold using ART. We filtered the resulting variant calls to exclude those supported only by data from the error-prone tails of sequencing reads as well as those that occured in genes containing repetitive regions - labelled as **FINAL output**. We collated all the data across all the simulations (white circles). Dotted lines represent expected frequencies for an allele for a given number of reads supporting the alternative base. Black dashed line: 1.0% allele frequency, Red dashed line: 1.5% allele frequency.

technical limitations and we should therefore devise a filtering step to remove all of them.

We analysed the outputs by mapping them to the genome of *M. tuberculosis* strain CCDC5079 (GenBank: CP001641) and calling the variants as for our patient samples. We found that each simulation generated approximately 25 v-SNPs. The distribution of frequencies and coverages at each variable locus was multimodal showing a great amount of variation. Filtering for repetitive regions and read-end bias simplified the frequency distribution and gave rise to a leptokurtic distribution with a very strong positive skew with all the variants occuring below the frequency of 1%. The coverage distribution was similarly simplified, resulting in a gaussian distribution, centered around 860-fold coverage. By exploring the relationship between variant frequency and read coverage for filtered v-SNPs resulting from simulation (see **Figure S2**) we observed that all variation fell on discrete integer error lines. This is a clear indication that the addition of an empirical frequency cut-off would greatly reduce platform-specific spurious minor variants. Specifically introducing a frequency cut-off of 1% would remove all minor variants resulting from sequencing errors in the coverage range we have within our study.

2.3 Culture-induced variation

We attempted to quantify the extent to which *in vitro* expansion modifies the population profile of *M. tuberculosis*. To this end, we picked two separate colonies and cultured them, extracted DNA from each culture and prepared two separate sequencing libraries for each DNA extraction. We sequenced the four resulting libraries to an average depth of approximately 1200-fold and called minor variants using the steps described in **section 2.1**. Discrepancies between pairs of libraries prepared from a single DNA extraction would provide with data that were analogous to the simulations discussed above. On the other hand any minor variants we observe that do not appear to be sequencing artefact should therefore represent biological variation arising from culture.

Following the removal of repetitive regions and variant calls with read-end bias we identified 288 variable sites across the four sequenced libraries (see table). As with simulated reads, we plotted the relationship of variant frequency and the coverage (see **Figure S3**). Echoing our observations with simulated reads, we found the majority of the v-SNPs to fall on discrete integer error lines for single colonies as well. These therefore match the error profile of sequencing artefacts and should be disregarded. Importantly we also observe a signature of culture expansion changing the profile of the population. Applying an empirical frequency cut-off of 1.5% reduces the overall rate of false discovery to less than 5% (6 vs 288 v-SNPs).

Five of the six remaining v-SNPs seem to have a comparatively low coverage.

From past experience in our labs, regions of the genome that have a comparatively low coverage tend to correspond to loci with problematic read mapping. We therefore performed a manual inspection of the v-SNPs and observed that two pairs of v-SNPs were very close to each other (3 and 183 bp distance) pointing to a potential sequencing artefact. A formal model to define a sequencing coverage cut-off for each v-SNP is beyond the scope of this paper, we therefore chose to apply a 1.5% frequency cut-off as the final post-call filtering step to the analysis of patient derived sequences.

v-SNP counts in single colony libraries

	Frequency cut-off		
Library ID	None	1.0%	1.5%
76-K26	116	4	1
76-K27	133	4	2
91-K28	22	6	1
91-K29	17	4	2

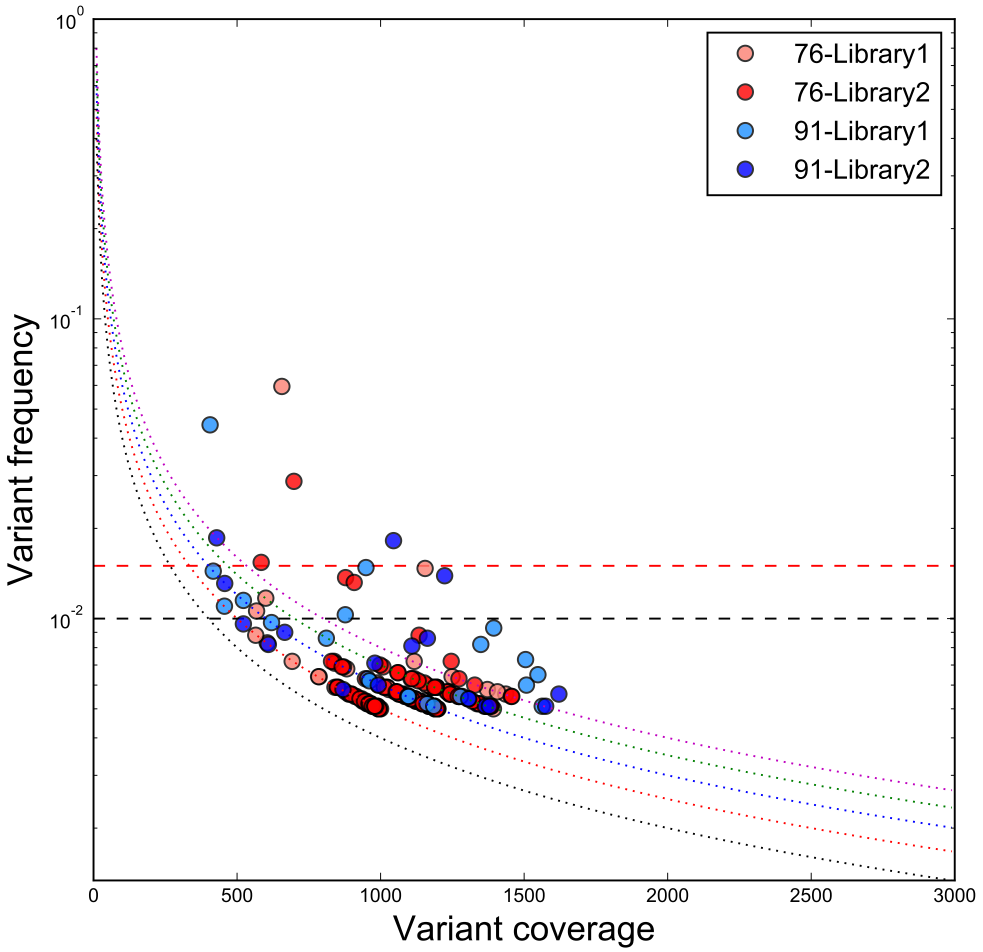

Figure S3: Culture-induced minor variants in MTBC. We picked two MTBC colonies ("76" and "91"), expanded them in culture, extracated DNA and generated two sequencing libraries from each DNA sample. We processed the resulting sequenicng data as described in **Section 2.1**. Errors resulting from sequencing artefacts (see **Figure S2**) show a frequency distribution that falls on discrete error lines (dotted lines). The majority of the minor variants detected in single colony samples mirror this distribution. The empirical frequency cut-off of 1.0% (black dashed line) does not sufficiently well curtail the variability within the samples. A more stringent 1.5% frequency cut-off (red dashed line) excludes the majority (283/288) of spurious variation that may influence our analysis.

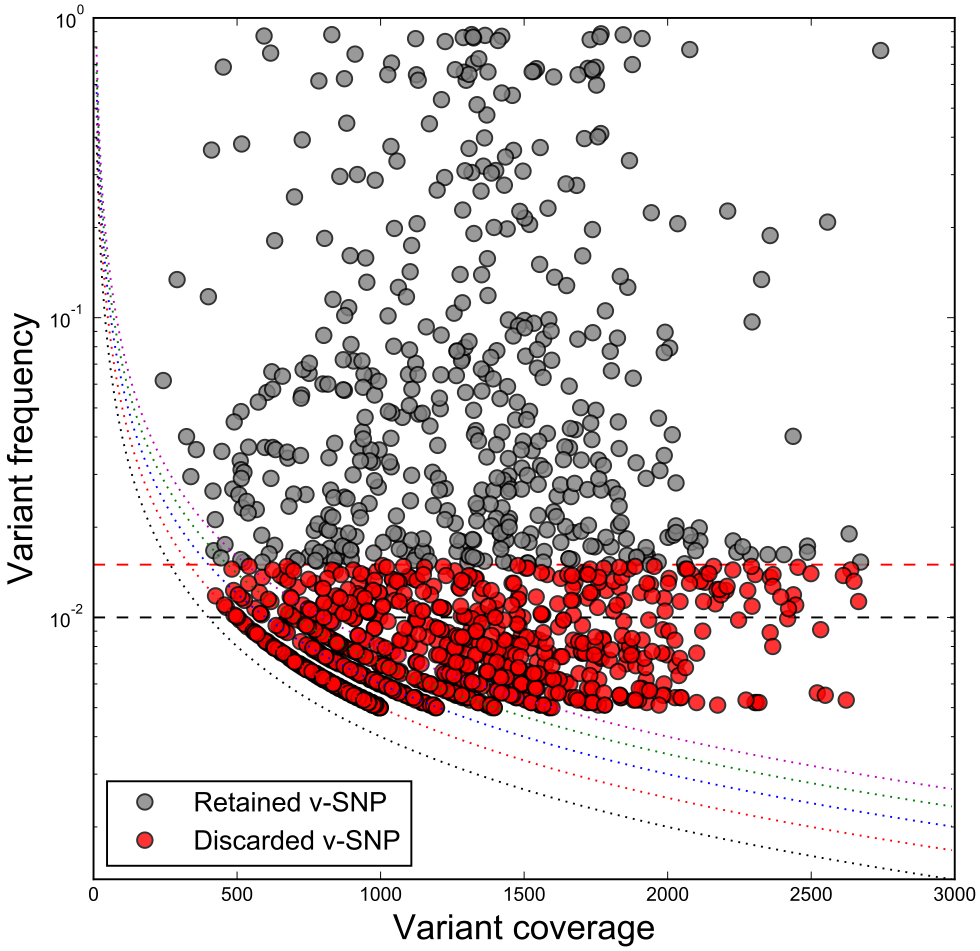

Figure S4: Retained minor variants in our patient population. v-SNPs that were retained for further analysis are shown in grey, while excluded v-SNPs are shown in red. The distribution of retained v-SNPs markedly contrasts that of variants obtained through *in silico* simulations (see **Figure S2**) and those derived from the *in vitro* expansion of single colonies shown in **Figure S3**.

3 Demographic indicators of bacterial killing

Given that all but two of the patients included in our study successfully achieved clinical cure, we reasoned that we should be able to recapitulate the clinical outcome by looking at the dynamics of the bacterial population. In order to define the expected dynamics that would arise from efficacious treatment we turned to in silico forward-time simulations of bacterial populations (6). We constructed a demographic model that would recapitulate key aspects of the natural history of MTBC infection - see **Figure S5A**. We expect that MTBC underwent an exponential expansion, following the initial infection, until it reached a carrying capacity and a density conducive to the onset of clinical symptoms (7). The population should then undergo a treatment-induced exponential decrease (8). In this context, each sputum sample represents a sampling bottleneck that is followed by a second phase of exponential growth as bacteria are grown in culture vials in preparation for genomic DNA extraction. We wanted to simulate the simplest scenario where the dynamics of rare alleles are governed entirely by chance so that any observed deviations in our data from the predicted trends would imply the involvement of underlying evolutionary processes. The simulation is based on the segregation of two alleles at a single genetic locus. In our scenario the two alleles had the same relative fitness but different starting frequencies - a rare allele at 5% and a frequent allele at 95%. The introduction of additional alleles or additional segregating loci was prevented by not allowing mutational events. We consider the outcome of each simulation to be analogous to the fate of an allele within the population. We varied the time at which virtual sampling occurred as illustrated in **Figure S5A** to interrogate the temporal dynamics within our system. The analysis of the trends across all of our simulations provided a context similar to that of a bacterial population within a host over time.

We focused on two parameters of the population: the *frequency of persistent alleles* and *heterozygosity*. Heterozygosity H was defined as in (9) and was calculated as:

$$h_l = 1 - \sum_{i=1}^{m} f_{il}^2 \tag{i}$$

$$H = \frac{1}{L} \sum_{l=1}^{L} h_l \tag{ii}$$

Where h_l is the heterozygosity at locus l, f_{il} is the frequency of the i-th allele at locus l, m is the number of possible alleles (A,C,G,T) and L is the size of the genome. Heterozygosity is calculated for each locus first as shown in Equation i and then calculated for the whole genome as shown in Equation ii.

The values of the parameters were calculated by resampling the population of interest with replacement (bootstrapping) 1000-times and then plotting the mean of the calculated outcomes together with the empirical 95% confidence interval which we define with the 25th and 975th permille of the re-sampled results. In the case of the mean allele frequencies, we performed a ordinary linear regression of the log_2 of the mean frequency over time or generation.

We observed that, according to expectation, the heterozygosity of the simulated population decreases over time, while the mean frequency of the rare alleles, in cases where the rare allele was present at the end of the simulation, increases over time. The demographic dynamics in MTBC populations from patients treated with at least 4 effective drugs followed the same trends, while those from patients treated with fewer than 4 effective drugs did not (see **Figure S5B-E**). We therefore conclude that our measured dynamics are biologically relevant and reflect the expected trends of bacterial populations undergoing extinction. Interestingly we observed that the relationship between the log_2 of the mean allele frequency and time/generations is exponential in the case of the simulations while it appears to be linear in the case of the treated patients. This may be an indication of the fact that the rate of killing within patients does not follow the dynamics of exponential decay, but rather decreases at a slower rate.

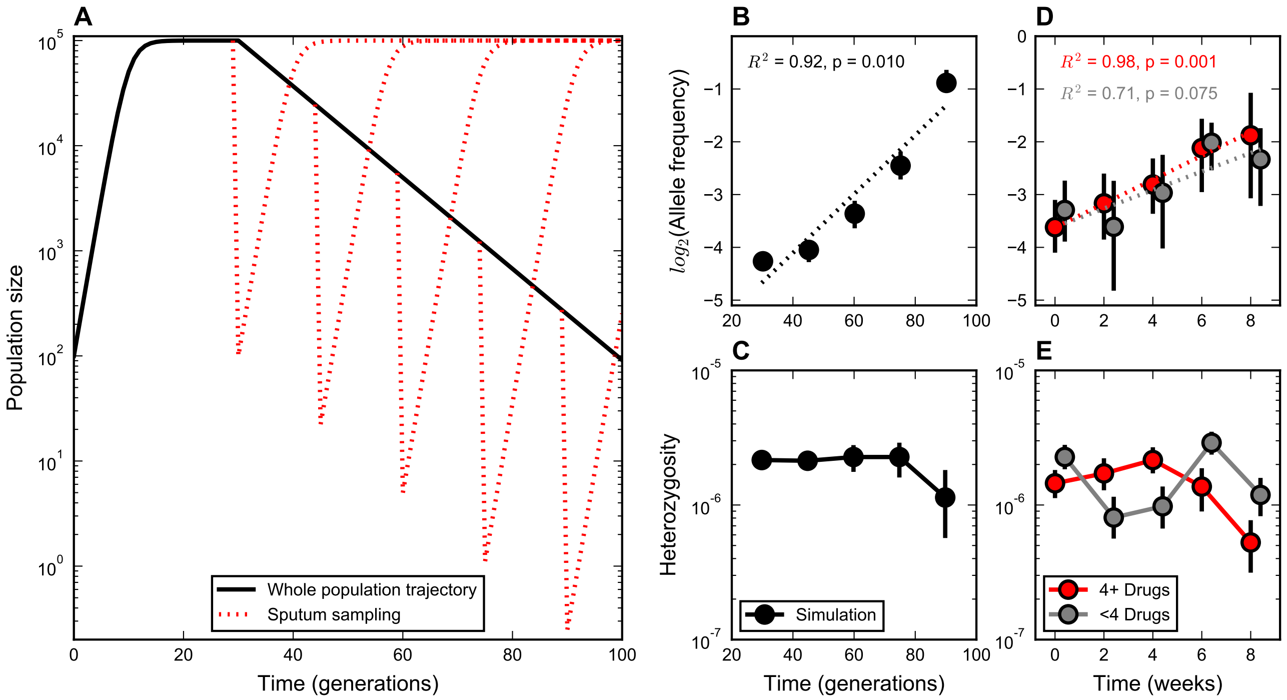

Figure S5: Demographic trends of MTBC populations within the host are consistent with bacterial killing. Demographic model used for simuPOP (6) forward time simulations of haploid populations (**A**). Mean allele frequencies of persistent minor variants following population simulations (black, **B**) or observed within patients treated with more than 4 ("4+ Drugs", red, **D**) or fewer than 4 effective drugs ("<4 Drugs", grey, **D**) treated patients. The means were obtained from 1000-fold resampling with replacement of simulated data. Error bars indicate the empirical 95% confidence interval $CI^{95\%}$ calculated from the resampled data. The R^2 and p-value of an ordinary linear regression are shown, and the regression line is indicated (dotted). Mean heterozygosity (9) of simulated populations (black, **C**), or populations within patients treated with more than 4 ("4+ Drugs", red, **E**) or fewer than 4 drugs ("<4 Drugs", grey, **E**). The means and CI $^{95\%}$ were obtained by re-sampling with replacement.

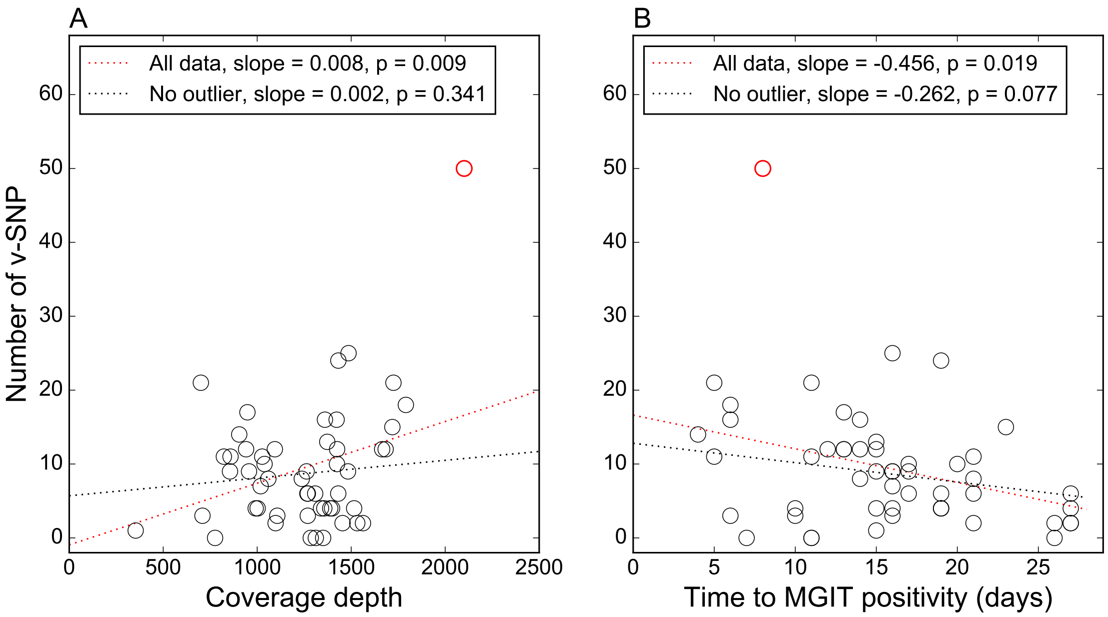

Figure S6: Sequencing depth does not bias v-SNP detection We observed a small but significant positive correlation between the number of v-SNPs and sequencing coverage (1 v-SNP per 100-fold sequence coverage, p=0.009). However, this correlation depended on a single outlier and was lost once we excluded it (2 v-SNPs per 1000-fold coverage, p=0.341). We also observed that the number of v-SNPs decreased slightly with increasing time to culture positivity (a proxy for decreasing bacterial burden). Considering all the data we calculated a loss of 0.5 v-SNPs per day of delay in culture positivity (p=0.019). Excluding the aforementioned outlier lead to a deterioration of the correlation: 0.3 v-SNPs per day of delay in culture positvity, p=0.077. The excluded outlier is depicted as the red datapoint.

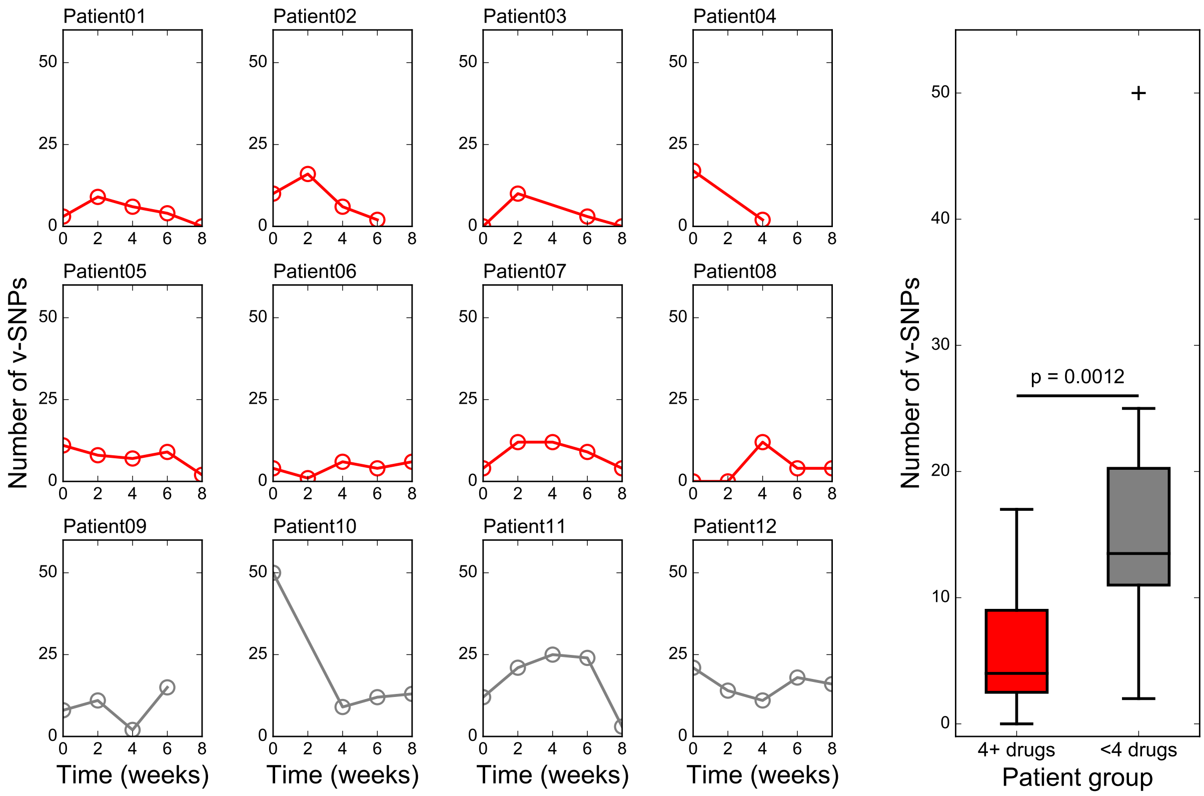

Figure S7: The number of detected v-SNPs for each patient varies over time. We show the collated data of v-SNP calls for each patient group in the boxplot. p-value derived from a Mann-Whitney U-test.

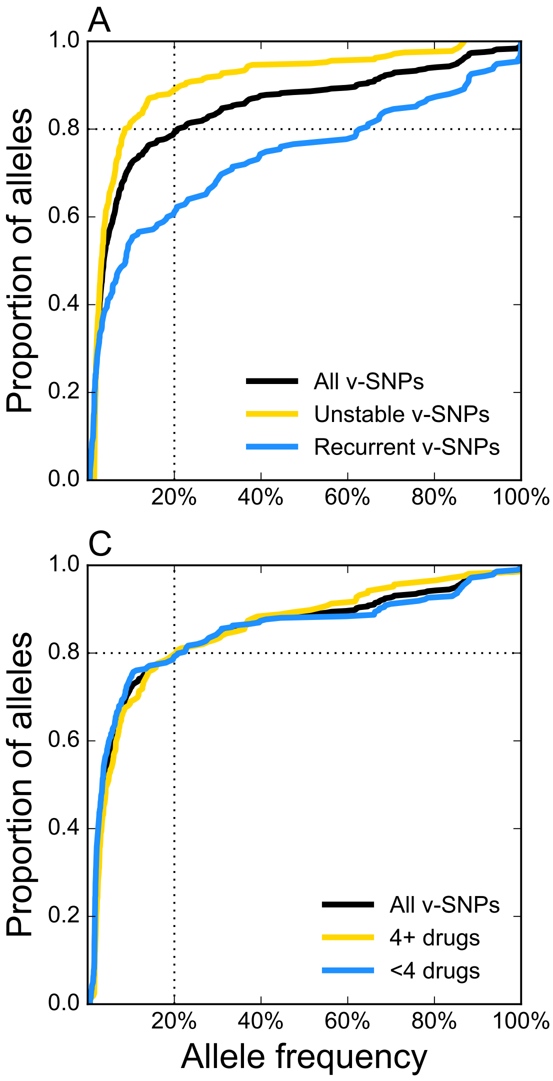

Figure S8: Cummulative distributions of allele frequencies. v-SNP data were collated across all time points and patients and grouped based on stability (recurrent vs unstable, **A**), translational impact (synonymous [SYN] vs nonsynonymous [NSY], **B**), regimen composition (more than four or fewer then four effective drugs, **C**) and treatment (pre-treatment vs various times post-treatment, **D**). The arrow in **D** indicates the temporal directionality in the shift of the distribution.

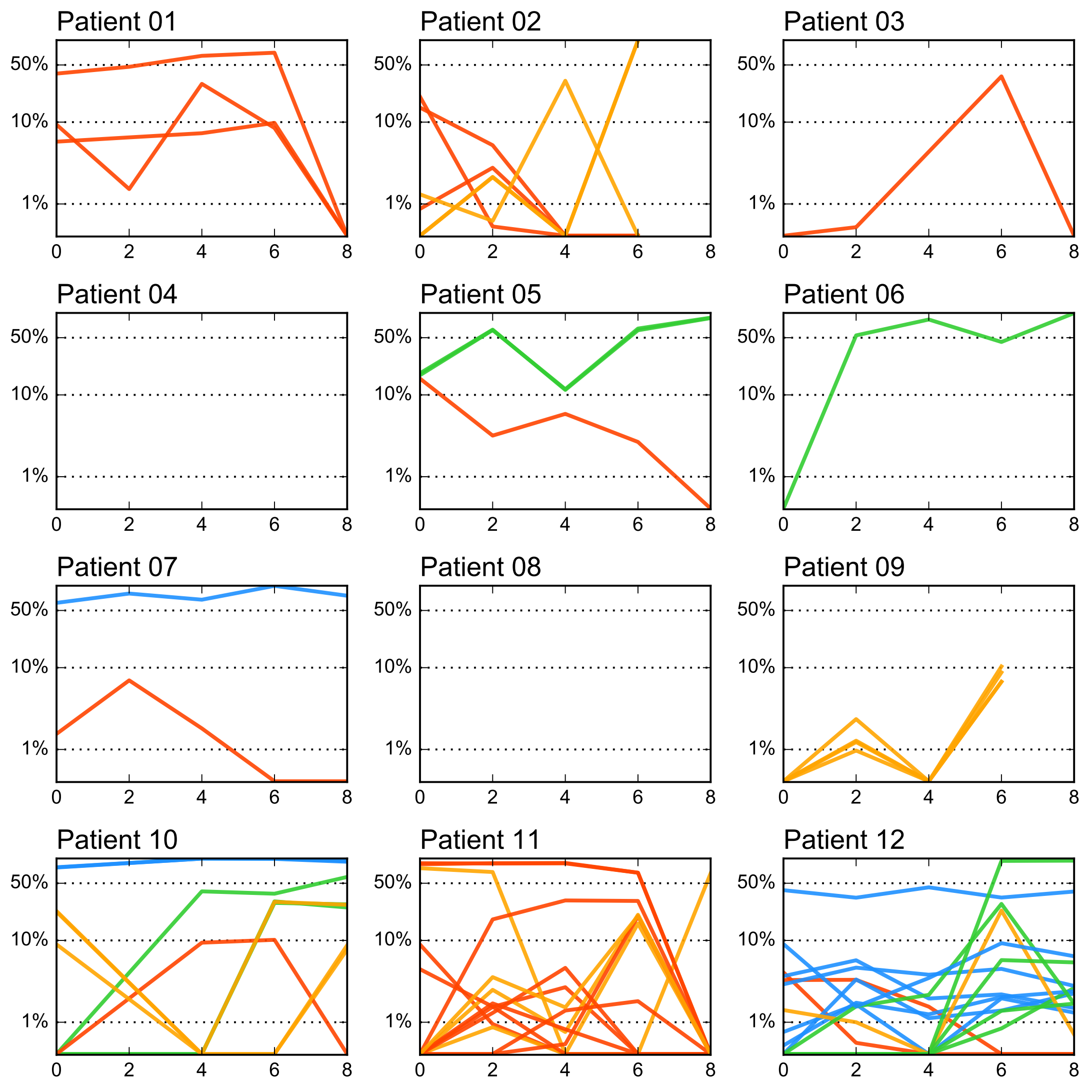

Figure S9: Frequency trajectories of recurrent alleles vary, showing a dynamic and heterogeneous population. Allele trajectories are colour-coded in a way that reflects Figure 2D in the main text. Green: ascending dynamics, Red: descending dynamic, Blue: Stable, Orange: sporadic.

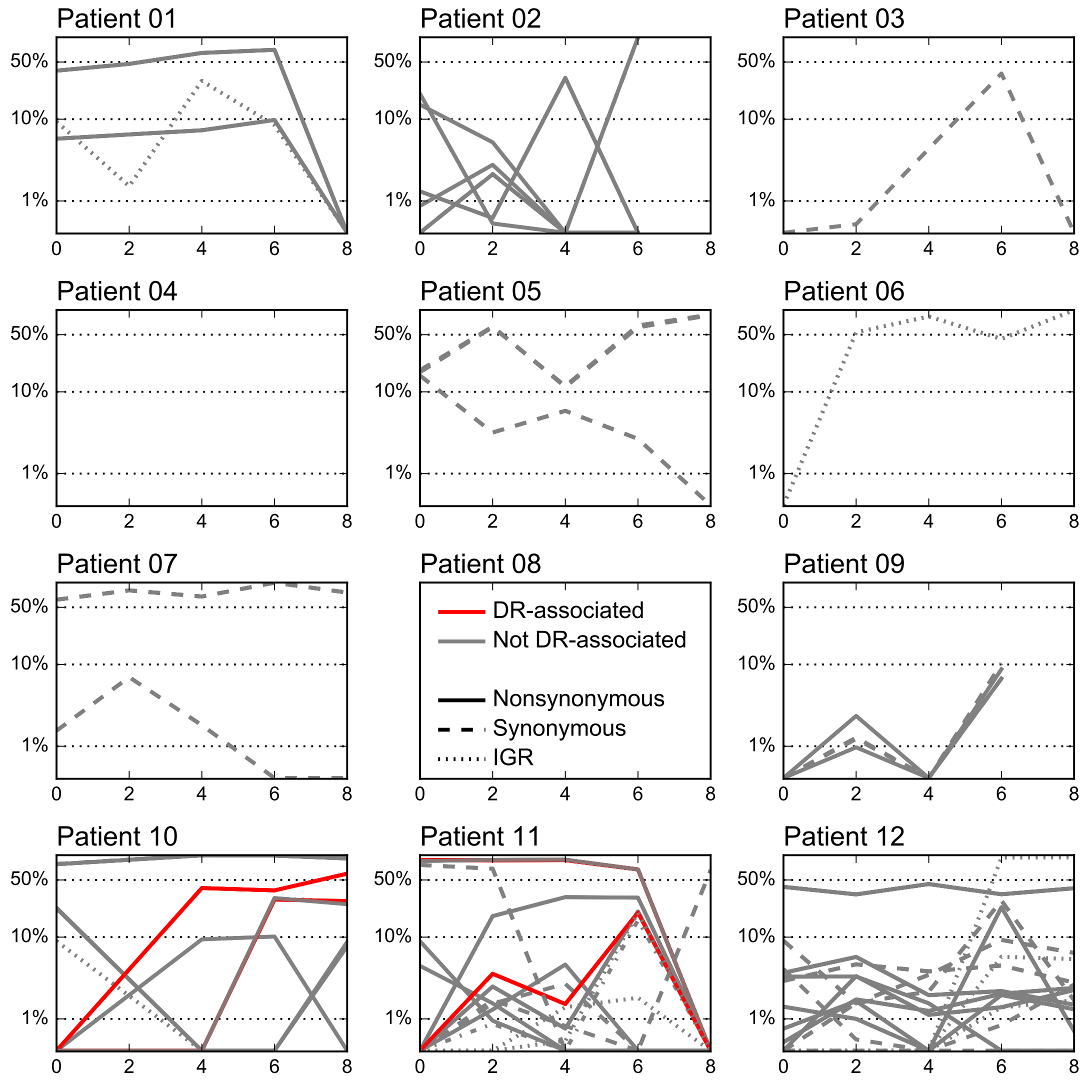

Figure S10: Sweeps of v-SNPs occur independently of the type of v-SNP affected the association of v-SNPs with drug resistance. The trajectories of recurrent alleles are shown for each patient. The style of the lines reflect v-SNP type and the colour reflects its association with drug resistance (drug resistance associated: red, not drug resistance associated: grey). We observed sweeps of nonsynonymous (continuous), synonymous (dashed) and v-SNPs in intergenic regions (IGR, dotted) in different patients. In patients 04 and 08 we did not detect any recurrent v-SNPs. Patients 02, 05, 09, 10, 11, 12 show evidence of shared trajectories of v-SNP combinations suggestive of different subpopulations within the patient lungs. Patient 10 provided the only example of a sweep to fixation involving a drug resistance allele. None of the other sweeps within the population were driven by drug resistance.

4 Phylogenetic analysis of patient isolates

4.1 Methodology

We collated 13,359 fixed SNPs (>95%) called against the CCDC5079 genome from 42 MTBC isolates. These included the 12 clinical isolates in this study and 30 representative MTBC isolates from publicly available databases (11). The concatenated sequences of the 42 isolates were used to generate a Maximum Likelihood (ML) phylogeny tree with MEGA6 (12). We used the Tamura-Nei nucleotide substitution model for phylogeny reconstruction, excluding nucleotide positions with gaps/missing data in more than 30% of the aligned taxa. We tested the robustness of the phylogeny with 500 bootstrap replications. The phylogeny tree was further visualized in FigTree(v1.4.2) - see **Figure S11**.

4.2 Majority of study isolates belong to the Beijing family

Using the charcterisation of sublineages defined previously (13) we determined that the reference strain CCDC5079 mutation belongs to the 2.2.1 sublineage of Lineage 2. This classification is based on the presence of 2505070 GA (equivalent to 2505085 GA in H37Rv numbering) and 796659 CT (equivalent to 797736 CT in H37Rv numbering) In addition to these SNPs, it also carries deletions RD105, RD207 and RD181, all of which are consistent with the classification. The majority (10/12) of the strains from our patients belong to the same sublineage as CCDC5079. The only exceptions are the strain from Patient11, which belongs to sublineage 2.2.2 – based on the presence of 345104 GT (equivalent to 346693 GT in H37Rv numbering). Unlike other patients, Patient06 was infected with a Lineage 4 strain. This strain carries SNPs 4310589 GA (equivalent to 4307886 GA in H37Rv numbering) and 4249207 GA (equivalent to 4246508 GA in H37Rv numbering) which identify it as a member of sublineage 4.4.2.

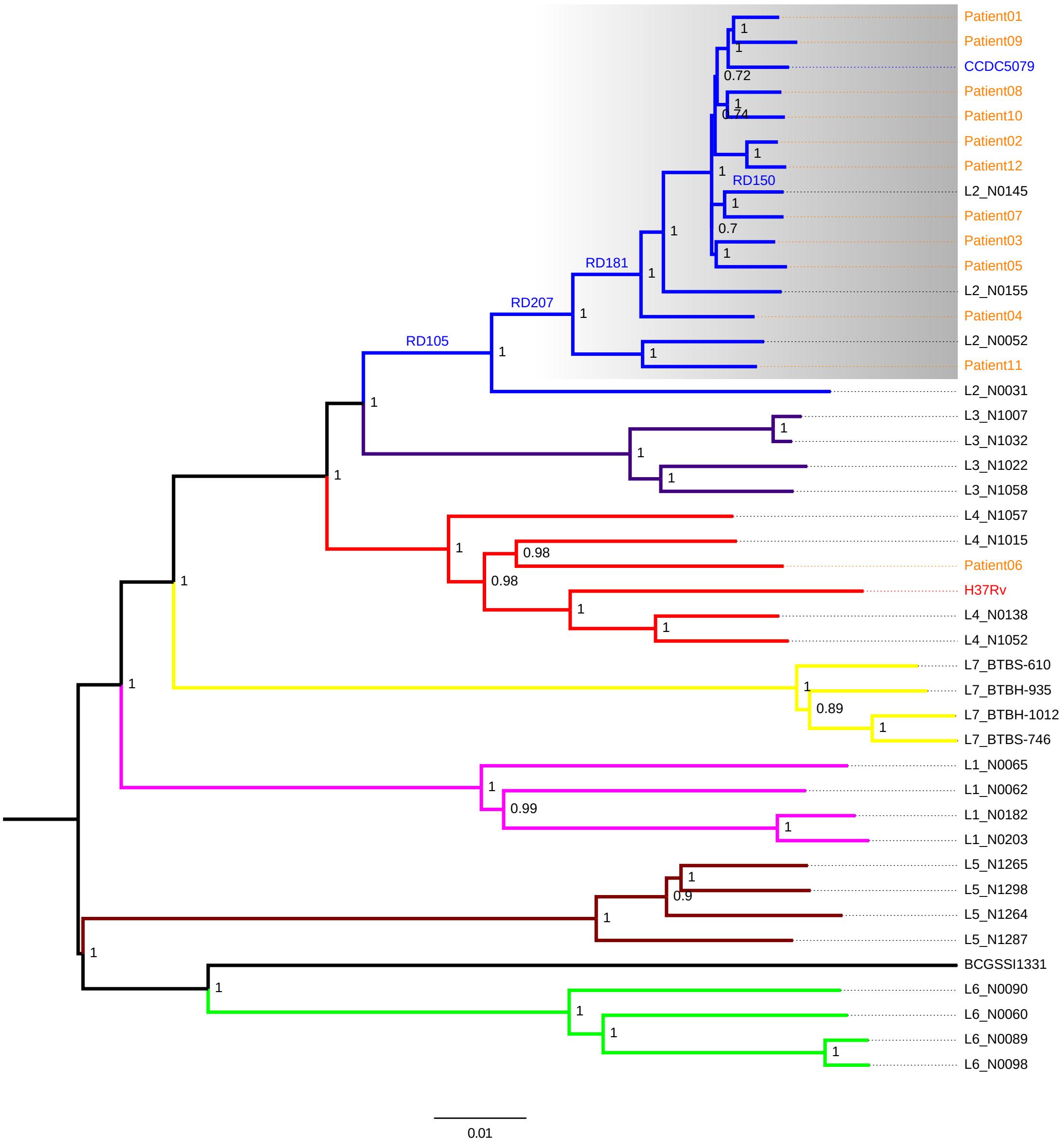

Figure S11: Most patients are infected with strains of the Beijing family. 13,359 f-SNPs were used to generate the phylogeny of 42 MTBC strains. We used a maximum likelyhood approach, with the node labels indicating the support values (proportions) for each branch based on 500 bootstraps. The trees were generated using MEGA6 (12) and rooted using the midpoint of the genetic distances. Reference strains CCDC5079 and H37Rv are highlighted in blue and red respectively, while the isolates from individual patients included in our study are shown in orange. Isolates belonging to the Beijing family of strains are highlighted by gray shading. Genomic deletions (RD) in Lineage 2 are marked on the branch in which they occur.

5 Supplementary datasets

All the data and analysis scripts used during this project are available from:

- DATA: zenodo doi: **10.5281/zenodo.322377**. Open licence: CC-BY-SA
- SCRIPTS: zenodo doi: **10.5281/zenodo.345135**. Open licence: GPL 3.0 **https://github.com/swisstph/TBRU_serialTB/**

A subset of these, containing summarised data are included with the manuscript - see the descriptions below.

5.1 Patient data

All clinical patient data are given in **Table S6 in Additional_file_2.csv** also available as "PATIENT_data.csv" on the repository. The categories in this file are coded as follows:

- **DRUG**: Drug susceptibility profile of patient
- **PATIENT_ID**: Patient ID
- **TIME**: time post treatment commencement (weeks)
- **TIME_TO_POSITIVITY_MGIT**: Week at which MTBC populations were first harvested from MGIT tubes
- **SEQUENCED_CULTURE**: Source of sequenced population (Culture conditions)
- **DEPTH**: Average read depth for the sample
- **NON_EFFICACIOUS**: Boolean classifier reflecting treatment efficacy (4+ drugs: 0, $<$4 drugs: 1)
- **vSNP_COUNT**: number of v-SNPs at timepoint
- **fSNP_COUNT**: number of f-SNPs at timepoint
- **EXPECTED_NSY**: expected number of nonsynonymous mutations based on mutated codons, substitution tables based on (10) (0 if insufficient data)
- **EXPECTED_SYN**: expected number of synonymous mutations based on mutated codons, substitution tables based on (10) (0 if insufficient data)

- **NEUTRAL_NSY**: simulated number of nonsynonymous mutations based on given mutated codons under the assumption of neutrality, substitution tables based on (10) (0 if insufficient data)
- **NEUTRAL_SYN**: simulated number of synonymous mutations based on given mutated codons under the assumption of neutrality, substitution tables based on (10) (0 if insufficient data)
- **OBSERVED_NSY**: observed number of nonsynonymous mutations
- **OBSERVED_SYN**: observed number of synonymous mutations
- **pNS**: calculated pNS from observed mutations (empty if insufficient data)
- **pNS_NEUTRAL**: calculated pNS from mutations simulated by assumption of neutrality, substitution tables based on (10) (empty if insufficient data)

5.2 v-SNP data

All collected and calculated data for all v-SNPs are given in the **Table S7 in Additional_file_2.csv** also available as "ALLELE_data.csv" on the repository. The categories in this file are coded as follows:

- **PATIENT_ID**: Patient ID
- **RESISTANCE**: Drug susceptibility profile of patient
- **FREQUENCY**: v-SNP frequency
- **LOG_FREQ**: $\log_2$ of v-SNP frequency
- **GENE**: ID tag of gene affected by v-SNP (H37Rv, CDS only)
- **LOCUS**: genomic locus of v-SNPs
- **TRANSITION**: Boolean classifier reflecting weather the mutation is a transition
- **WT_CODON**: wild type codon affected by mutation
- **TIME**: time post treatment commencement (weeks)
- **SNP_TYPE**: v-SNP type

- **NON_EFFICACIOUS**: Boolean classifier reflecting treatment efficacy (4+ drugs: 0, <4 drugs: 1)
- **RECURRENT**: Boolean classifier reflecting whether a v-SNP is recurrent

5.3 f-SNP data

All collected and calculated data for all f-SNPs are given in the **Table S8 in Additional_file_4.csv** also available as "FIXED_ALLELE_data.csv" on the repository. The categories in this file are coded as follows:

- **PATIENT_ID**: Patient ID
- **RESISTANCE**: Drug susceptibility profile of patient
- **GENE**: ID tag of gene affected by v-SNP (CCDC5079, CDS only)
- **LOCUS**: genomic locus of v-SNPs
- **TRANSITION**: Boolean classifier reflecting weather the mutation is a transition
- **WT_CODON**: wild type codon affected by mutation
- **SNP_TYPE**: v-SNP type
- **NON_EFFICACIOUS**: Boolean classifier reflecting treatment efficacy (efficacious: 0, non-efficacious: 1)

5.4 Predefined Genesets

Genesets used for mutation enrichment analysis are listed in **Table S9 in Additional_file_5.csv** also available as "PREDEFINED_gene_sets.csv" on the repository. The categories in this file are coded as follows:

- **GENE**: ID tag of gene (H37Rv, CDS only)
- **GENE_SET**: Gene set to which the gene belongs
- **REFERENCE**: Reference for the gene set
- **EXCLUDED**: Number of transitions considered/resampled

References

[1] T. D. Lieberman, K. B. Flett, I. Yelin, T. R. Martin, A. J. McAdam, G. P. Priebe and R. Kishony (2014) *Genetic variation of a bacterial pathogen within individuals with cystic fibrosis provides a record of selective pressures.* Nat Genet, 46:82–87.

[2] M. Coscolla, *et al.* (2015) *M. tuberculosis T Cell Epitope Analysis Reveals Paucity of Antigenic Variation and Identifies Rare Variable TB Antigens.* Cell Host Microbe 18:538–548.

[3] D. C. Koboldt *et al.* (2012) *VarScan 2: somatic mutation and copy number alteration discovery in cancer by exome sequencing.* Genome BIol 3:568–576.

[4] A. Wilm *et al.* (2012) *LoFreq: a sequence-quality aware, ultra-sensitive variant caller for uncovering cell-population heterogeneity from high-throughput sequencing datasets.* Nucleic Acids Res 40:11189–11201.

[5] W. Huang, L. Li, J. R. Myers and G. T. Marth (2012), *ART: a next-generation sequencing read simulator.* Bioinformatics 28:593–594.

[6] B. Peng and M. Kimmel (2005), *simuPOP: a forward-time population genetics simulation environment.* Bioinformatics 21:3686–3687.

[7] S. D. Lawn, R. Wood and R. J. Wilkinson (2010) *Changing concepts of "latent tuberculosis infection" in patients living with HIV infection.* Clin Dev Immunol 2011:980594.

[8] A. Jindani, V. R. Aber, E. A. Edwards and D. A. Mitchinson (1980) *The early bactericidal activity of drugs in patients with pulmonary tuberculosis.* Am Rev Respir Dis 121:939–949.

[9] J. M. Cuevas, A. Willemsen, J. Hillung, M. P. Zwart and S. F. Elena (2015) *Temporal dynamics of intrahost molecular evolution for a plant RNA virus.* Mol Biol Evol 32:1132–1147.

[10] M. Hasegawa, H. Kishino, and T. Yano (1985) *Dating of the Human-Ape Splitting by a Molecular Clock of Mitochondrial DNA* J Mol Evol 22:160–174.

[11] I. Comas *et al.* (2013) *Out-of-Africa migration and Neolithic co-expansion of Mycobacterium tuberculosis with modern humans* Nat Genet 45:1176–1182.

[12] K. Tamura, G. Stecher, D. Peterson, A. Filipski, and S. Kumar (2013) *MEGA6: Molecular Evolutionary Genetics Analysis Version 6.0* Mol Biol Evol 30:2725–2729.

[13] F. Coll *et al.* (2014) *A robust SNP barcode for typing Mycobacterium tuberculosis complex strains* Nat Commun 5:4812 doi:10.1038/ncomms5812.
